# Effectiveness of Integrated Care for Diabetes Mellitus Type 2, Cardiovascular and Chronic Respiratory Diseases: A Systematic Review and Meta-Analysis

**DOI:** 10.5334/ijic.7744

**Published:** 2024-08-19

**Authors:** Pim P. Valentijn, Liza Tymchenko, Wiro Gruisen, Bem Bruls, Fernando Abdalla Pereira, Rosa Y. Arends

**Affiliations:** 1 Essenburgh Research & Consultancy, Essenburgh Group, Harderwijk, the Netherlands; 2 Research Group Nursing, Hanze University of Applied Sciences, Groningen, the Netherlands; 3 Healthcare Division, CZ Health Insurance, Tilburg, the Netherlands; 4 Health Centre Hoensbroek North, Hoensbroek, the Netherlands; 5 General Practitioners Eastern South Limburg, Heerlen, the Netherlands; 6 Department of Health Affairs & Policy Research, Vivactis Weber, Madrid, Spain; 7 University of Applied Sciences Utrecht, Utrecht, the Netherlands

**Keywords:** Integrated care, Diabetes type 2, Cardiovascular risk management, COPD, Systematic review, Meta-analysis

## Abstract

**Introduction:**

In this paper, we use the Rainbow Model of Integrated Care (RMIC) framework to evaluate the effectiveness of integrated care in terms of enhancing the outcomes of chronic conditions such as diabetes mellitus type 2 (DMT2), cardiovascular diseases (CVD), chronic respiratory diseases (CRD), or their combinations.

**Methods:**

The data extracted from randomized controlled trials (RCT) of integrated care interventions for DMT2, CVD, and CRD (follow-up ≥ 3 months) in 11 databases were analysed using random-effects meta-analysis.

**Results:**

A total of 54 eligible studies covering 12,976 participants, with a mean follow-up of 54 weeks, were included. In moderate-quality evidence, integrated care interventions reduced mortality for CVD, adverse events for CVD and DMT2, and improved quality of life for CVD and DMT2, physical and mental functioning, self-management, and blood pressure control.

**Conclusion:**

Integrated care can reduce all-cause mortality, adverse events, and improve quality of life, physical and mental functioning, self-management and blood pressure control in chronic disease patients. However, available evidence for some outcomes (e.g., all-cause hospital admissions) remains uncertain.

## Introduction

Chronic diseases, broadly defined as conditions lasting one year or longer and requiring ongoing medical attention or limiting daily life activities, or both [1], are the leading cause of global mortality and disability and have considerable economic implications [2,3,4]. The most prevalent chronic diseases (e.g., CVD, DMT2) [3,4,5] are associated with an increased risk of premature death, disability, reduced quality of life, and rising healthcare costs, which makes them a prime global public health concern. The growing prevalence of chronic diseases prevents healthcare providers’ effective management of those conditions, resulting in fragmented care experience for patients, increased costs, and decreased resources available for other preventive services [6]. To address this concern, health systems must invest in integrated care strategies to reduce the burden on individuals and society as a whole [1,7].

The overarching goal of integrated care is three-fold: to improve health outcomes, enhance patient experiences, and make smarter use of available health service resources [8]. Enabling healthcare providers to develop holistic care plans focused on the patients’ physical, mental, and social needs, integrated care helps to coordinate and optimize care across different healthcare providers and settings [9]. Several studies reported moderate-quality evidence on a positive clinical benefit of integrated care in DMT2 management [10,11], CVD [12,13,14,15], and CRD [16,17]. However, due to variations in content, duration, and delivery of integrated care interventions, strong conclusions about the efficacy cannot be drawn [6,18].

**Figure 1:**
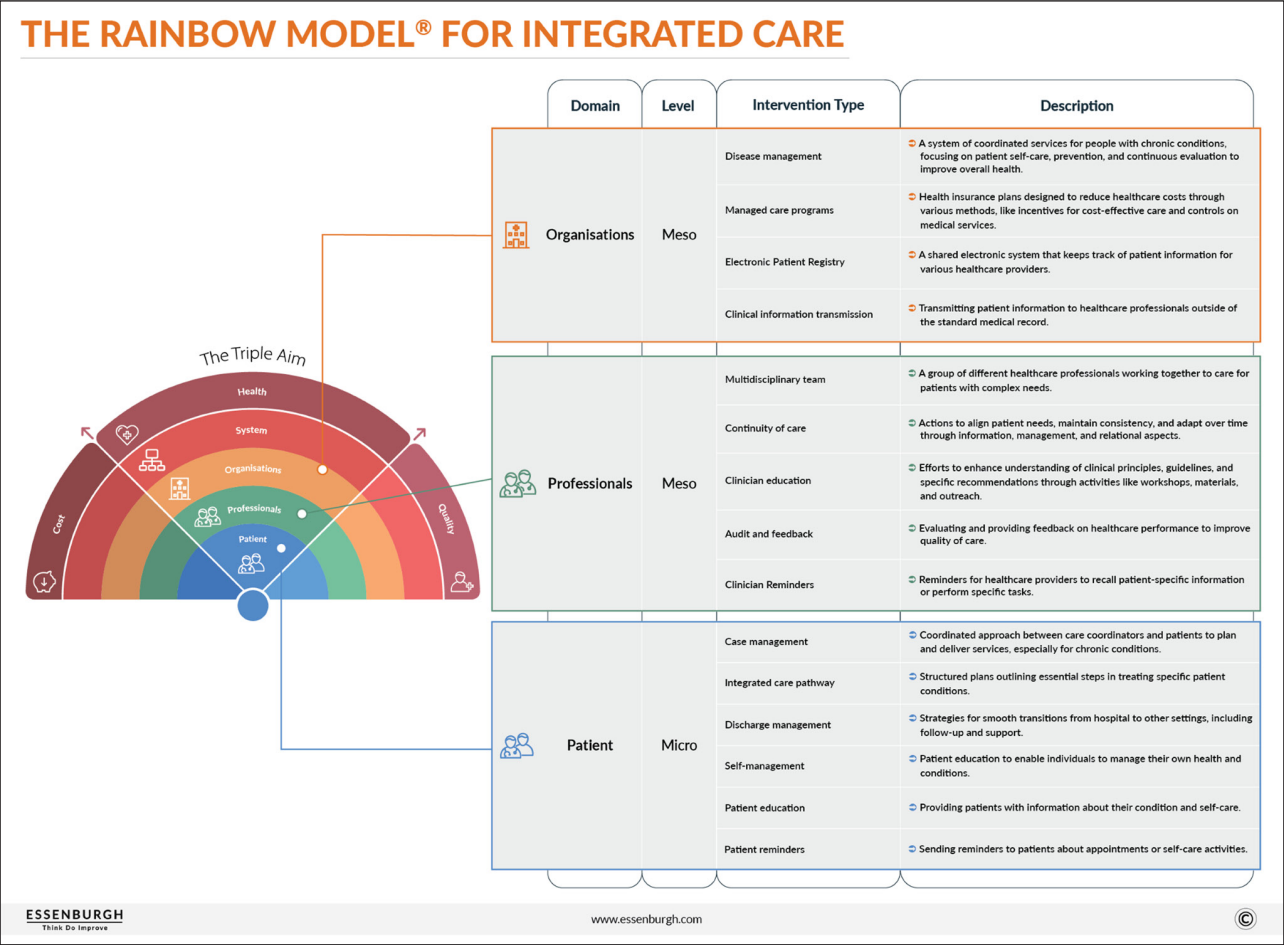
Rainbow model for integrated care [22].

One specific gap concerns which type of integrated care intervention, or combination thereof, is the most effective. To address this gap and inform policy decisions, we previously explored the multiple facets of integrated care using the Rainbow Model of Integrated Care (RMIC) [9,19,20] (Figure 1). Considering that the potential variability in effectiveness depends on specific combinations of interventions [8,21], identifying the optimal combination is essential for evidence-based decision-making. Extending our previous research [8], in this study, we use the RMIC framework to comprehensively analyse the effectiveness of (combinations of) integrated care interventions for DMT2, CVD, and CRD in published RCTs and evaluate variations in outcomes attributed to different types of integrated care interventions.

## Methods

Detailed methods of the conducted systematic review and meta-analysis followed the PRISMA guidelines and are described in the study protocol registered at the International Prospective Register of Systematic Reviews (registration number CRD42022311507; https://www.crd.york.ac.uk/prospero/).

### Literature search

We searched 11 electronic databases (e.g., PubMed, Google Scholar, Web of Science, etc.) using keywords related to DMT2, CVD, and CRD and integrated care from the earliest available date to August 2021 (Table 1 in Appendix I).

### Study selection

Two researchers (L.T. and A.F.) independently reviewed studies for eligibility, evaluated criteria, assessed risk of bias, and extracted relevant data. Disagreements were resolved through discussion, with the third author making a final decision if necessary. Eligibility included RCTs evaluating integrated care interventions with a follow-up of at least 3 months, participants aged over 18 years with specific conditions (Table 2 in Appendix I). Studies published in languages other than English were excluded.

### Data extraction and risk of bias assessment

Data extraction and risk assessment were conducted independently by two researchers (L.T. and A.F.) using Covidence software. Evaluated methodological risks included sequence generation, allocation concealment, and blinding of outcome assessors.

### Data synthesis and analysis

Primary outcomes of interest were all-cause mortality, all-cause hospital admissions, costs, adverse events, and healthcare use. Secondary outcomes included quality of life, physical functioning, weight management (i.e., Weight and Body Mass Index [BMI]), mental health, self-management, patient knowledge, and life-style behaviour (i.e., diet, smoking status) as measured by validated measures. Tertiary outcomes included biochemical measures such as HbA1C, cholesterol, pulmonary measures, and blood pressure control.

A combination of hierarchical agglomerative and non-hierarchical clustering methods was used to detect integrated care intervention clusters based on RMIC domains. The clusters were used to evaluate outcome differences among integrated care clusters [8,21]. Reliability of subgroup analyses was ensured only for groups comprising a minimum of 10 studies.

DerSimonian and Laird random-effects models were employed for continuous outcomes, reporting standardized mean differences (SMD) with 95% confidence intervals (CI). Binary outcomes were analysed using Mantel-Haenszel method, reporting risk ratios (RR) with 95% CI. Meta-analyses required a minimum of three independent studies to justify. Variability in treatment effects was evaluated using I2 statistics, and subgroup analyses explored intervention duration and setting. Sensitivity analyses excluded studies with high bias risk, long follow-up (≥ 12 months), or large sample size (> 200 participants). We used descriptive statistics to summarize the data, including means and standard deviations for continuous data and frequencies and percentages for binary data. For subgroup and sensitivity analysis, we used the test for subgroup differences provided in the R Studio package meta. All analyses were conducted using R Studio 2021.09.01 (Build 372) and the libraries dmetar, esc, tidyverse, meta, grid, robvis, pvclust, and factorextra.

### Quality of evidence

Quality of evidence for each pooled analysis was evaluated using the GRADE approach. Since perfect blinding is difficult to achieve in integrated care interventions, we did not lower the quality of evidence for performance and/or detection bias. Two researchers (L.T. and P.P.V.) independently rated evidence quality, resolving discrepancies through discussion.

## Results

### Search results and study characteristics

**Figure 2:**
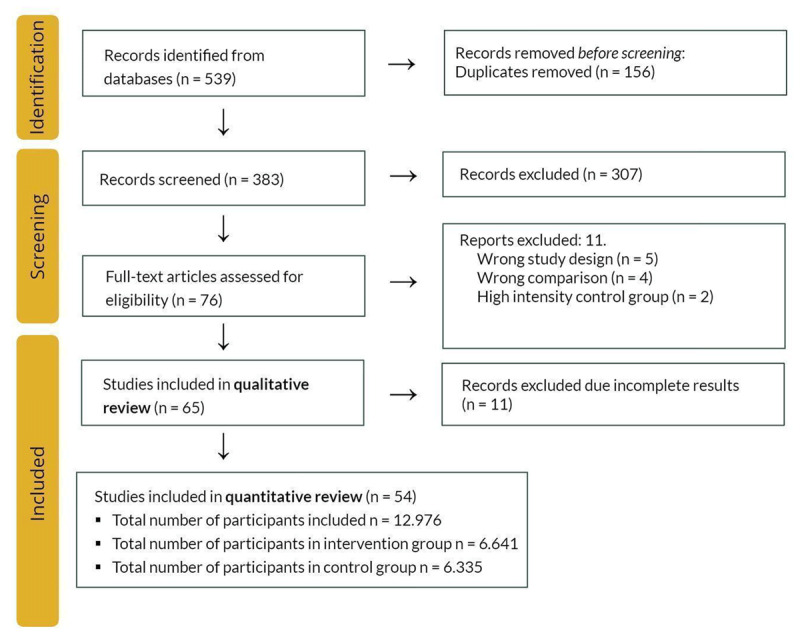
Flowchart of search strategy and study selection process.

A total of 539 publications were initially identified, with 383 selected for title and abstract screening after removing 156 duplicates. Subsequently, 76 publications were selected for full-text screening, of which 11 were removed due to incorrect study design, comparison, or high intensity control group. Thus, 65 studies were included in the qualitative review ([23,24,25,26,27,28,29,30,31,32,33,34,35,36,37,38,39,40,41,42,43,44,45,46,47,48,49,50,51,52,53,54,55,56,57,58,59,60,61,62,63,64,65,66,67,68,69,70,71,72,73,74,75,76,77,78,79,80,81,82,83,84,85,86,87], Tables 3–4 in Appendix I). After removing 11 further studies due to incomplete outcome reporting, 54 RCTs were retained in the final dataset (Figure 2).

### Intervention characteristics

Intervention characteristics (Tables 3–4 in Appendix I) revealed that most interventions were conducted in out-patient community care settings (46.30%), followed by transitional care settings (38.89%) and in-patient institutional (14.81%). Most targeted individuals had CVD (40.74%), followed by those with DMT2, (24.07%), COPD (12.96%), and multiple comorbidities (22.22%). Integrated care interventions primarily focused on patient (54 studies), professional (50 studies), or organizational levels (42 studies), with durations ranging from 1 to 216 months (median 6 months, average 12 months).

### Integrated care clusters

Two distinct clusters of integrated care interventions emerged across the 54 reviewed articles: “Patient empowerment” (22 studies) and “Network care coordination” (32 studies) (Table 1). The former focused mainly on patient and professional levels, predominantly in transitional care settings (54.55%), while the latter spanned patient, professional, and organizational levels, mostly in outpatient community care settings (50.00%). These clusters demonstrated significant variations in types of interventions used. Descriptive analysis of the cluster impact on outcomes is provided in Table 5 in Appendix I.

**Table 1:** Clusters of integrated care interventions [9].


RAINBOW MODEL INTERVENTIONS CHARACTERISATION	TOTAL STUDIES	CLUSTER 1: PATIENT EMPOWERMENT	CLUSTER 2: NETWORK CARE COORDINATION	CLUSTER DIFFERENCES

N (%)	54 (100%)	22 (41%)	32 (59%)	

**Organizational level**				

Disease management	34 (63%)	6 (27%)	28 (88%)	0.000***

Managed care programs	10 (19%)	4 (18%)	6 (19%)	1.000

Electronic patient registry	15 (28%)	1 (5%)	14 (44%)	0.002**

Clinical information transmission	7 (13%)	3 (14%)	4 (13%)	1.000

**Professional domain**				

Multidisciplinary team	41 (76%)	11 (50%)	30 (94%)	0.001***

Continuity of care	28 (52%)	6 (27%)	22 (69%)	0.005**

Clinician education	27 (50%)	13 (59%)	14 (44%)	0.41

Audit and feedback	9 (17%)	2 (9%)	7 (22%)	0.28

Clinician reminders	9 (17%)	2 (9%)	6 (19%)	0.45

**Patient domain**				

Case management	33 (61%)	1 (5%)	31 (97%)	0.000***

Integrated care pathway	24 (44%)	4 (18%)	20 (63%)	0.002***

Discharge management	15 (28%)	4 (18%)	11 (34%)	0.23

Self-management	26 (48%)	14 (64%)	12 (38%)	0.09

Patient education	25 (46%)	13 (59%)	12 (38%)	0.17

Patient reminders	3 (6%)	2 (9%)	1 (3%)	0.56


**Significant level < 0.01. ***Significant level < 0.001.

### Quality of included studies

**Figure 3:**
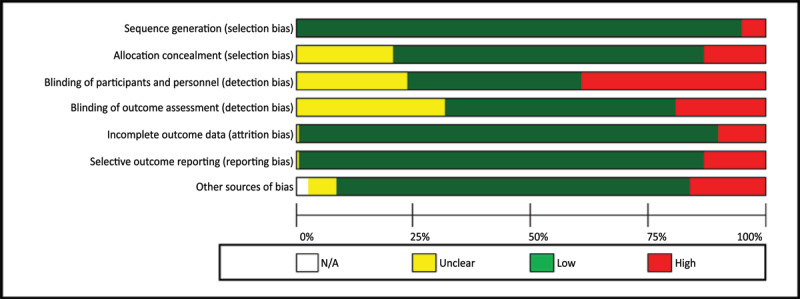
Summary risks of bias in included studies.

Figure 3 summarizes the risk of bias in included studies, with 71.96% demonstrating low risk, 13.23% unclear risk, and 14.81% high risk across quality items.

### Effect of integrated care interventions

#### Primary outcomes

##### All-cause mortality

**Figure 4:**
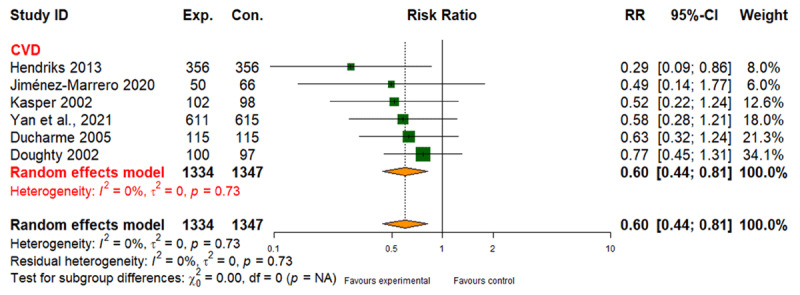
Effect of integrated care on all-cause mortality.

Six studies involving 2,681 participants revealed a 40% lower risk of mortality for CVD patients receiving integrated care (RR: 0.60; 95% CI: 0.44 to 0.81), with moderate evidence quality (Figure 4 and Table 2). No studies focused on DMT2 or CRD mortality (Table 6 in Appendix I).

**Table 2:** Summary of findings and assessment of quality of evidence for outcomes.


CERTAINTY ASSESSMENT							N OF PATIENTS		EFFECT		CERTAINTY
		
N OF STUDIES	STUDY DESIGN	RISK OF BIAS	INCONSISTENCY	INDIRECTNESS	IMPRECISION	OTHER CONSIDERATIONS	[INTERVENTION]	[COMPARISON]	RELATIVE (95% CI)	ABSOLUTE (95% CI)	

PRIMARY OUTCOMES											

All-cause mortality (follow-up: mean 39 weeks)											

6	randomised trials	serious^a^	not serious	not serious	not serious	none	56/1334 (4.2%)	97/1347 (7.2%)	RR 0.60 (0.44 to 0.81)	29 fewer per 1.000(from 14 fewer to 44 fewer)	⨁⨁⨁◯ Moderate

All-cause hospital admissions (follow-up: mean 39 weeks)											

6	randomised trials	serious^a^	serious^b^	not serious	not serious	none	429/1334 (32.2%)	659/1347 (48.9%)	RR 0.63(0.56 to 0.71)	181 fewer per 1,000 (from 142 fewer to 215 fewer)	⨁⨁◯◯ Low

Adverse events (follow-up: mean 45.5 weeks)											

4	randomised trials	not serious	serious^b^	not serious	not serious	none	143/1251 (11.4%)	177/1108 (16.0%)	RR 0.53(0.27 to 1.05)	75 fewer per 1.000(from 8 fewer to 117 fewer)	⨁⨁⨁◯ Moderate

Healthcare use (follow-up: mean 52 weeks)											

3	randomised trials	not serious	serious^b^	not serious	serious^c^	none	135	132	-	SMD 0.3 SD higher (0.05 lower to 1.10 higher)	⨁⨁◯◯ Low

**SECONDARY OUTCOMES**											

Quality of life (follow-up: mean 35 weeks)											

15	randomised trials	not serious	serious^b^	not serious	not serious	none	2278	2180	-	SMD 0.38 SD higher (0.03 higher to 0.73 higher)	⨁⨁⨁◯ Moderate

Physical functioning (follow-up: mean 34 weeks)											

19	randomised trials	not serious	serious^b^	not serious	not serious	none	2062	1843	-	SMD 0.17 SD higher (0.03 higher to 0.30 higher)	⨁⨁⨁◯ Moderate

Weight management (follow-up: median 45 weeks)											

10	randomised trials	serious^a^	serious^b^	not serious	serious^c^	none	902	693	-	SMD 0.08 SD higher (0.07 lower to 0.24 higher)	⨁◯◯◯ Very low

Mental health (follow-up: mean 35 weeks)											

20	randomised trials	not serious	serious^b^	not serious	not serious	none	2319	2083	-	SMD 0.29 SD higher (0.12 higher to 0.46 higher)	⨁⨁⨁◯ Moderate

Self-management (follow-up: mean 30 weeks)											

13	randomised trials	not serious	serious^b^	not serious	not serious	none	1487	1304	-	SMD 0.3 SD higher (0.10 higher to 0.50 higher)	⨁⨁⨁◯ Moderate

TERTIARY OUTCOMES											

HbA1c (follow-up: mean 40 weeks)											

15	randomised trials	serious^a^	serious^b^	not serious	not serious	none	947	865	-	SMD 0.45 SD higher (0.15 higher to 0.75 higher)	⨁⨁◯◯ Low

Cholesterol (follow-up: mean 52 weeks)											

6	randomised trials	serious^a^	not serious	not serious	serious^c^	none	590	376	-	SMD 0.02 SD lower (0.12 lower to 0.07 higher)	⨁⨁◯◯ Low

Pulmonary measures (follow-up: mean 264 weeks)											

4	randomised trials	serious^a^	not serious	not serious	serious^c^	none	713	785	-	SMD 0.17 SD higher (0.16 lower to 0.49 higher)	⨁⨁◯◯ Low

Blood pressure control (follow-up: mean 50 weeks)											

11	randomised trials	serious^a^	not serious	not serious	not serious	none	1601	1395	-	SMD 0.11 SD higher (0.00 to 0.21 higher)	⨁⨁⨁◯ Moderate


**CI:** confidence interval; **RR:** risk ratio; **SMD:** standardised mean difference. **Explanations**
Most of the studies have high frequency of other bias. Large heterogeneity between studies (I2>50%). 95% CI includes possible benefits from both control and health interventions.

##### All-cause hospital admissions

**Figure 5:**
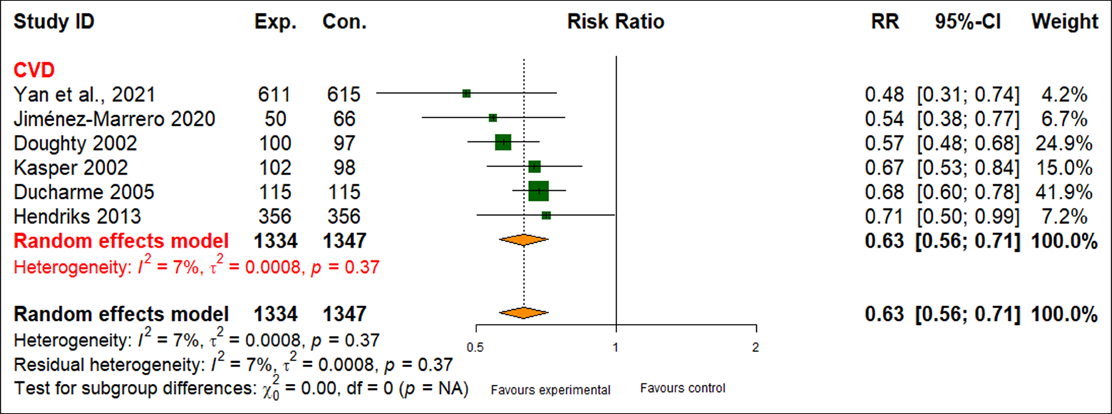
Effect of integrated care on all-cause hospitalization.

Integrated care recipients for CVD experienced a 37% lower risk of hospitalization (RR: 0.63; 95% CI: 0.56 to 0.71) based on low-quality evidence (Figure 5 and Table 2). No reduction in hospitalizations was observed for DMT2 or CRD (Table 6 in Appendix I).

##### Adverse events

**Figure 6:**
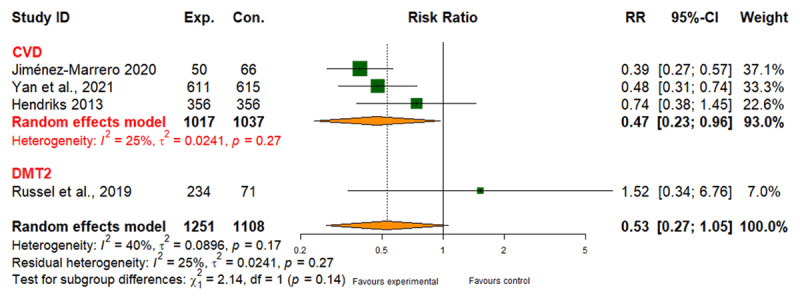
Effect of integrated care on adverse events.

Integrated care recipients exhibited a 47% lower risk of adverse events for CVD and DMT2 (RR: 0.53; 95% CI: 0.27 to 1.05), with moderate evidence quality (Figure 6 and Table 2). No reduction in adverse events was found for CRD (Table 6 in Appendix I).

##### Healthcare use

**Figure 7:**
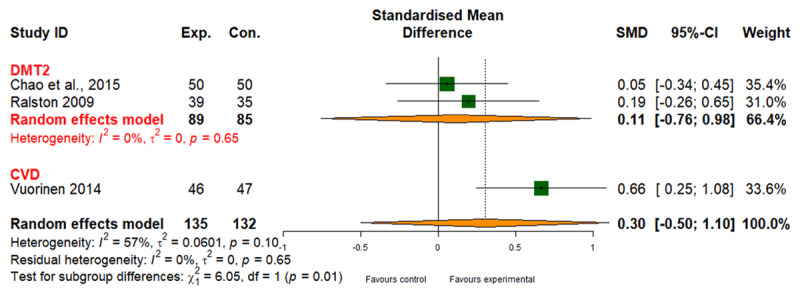
Effect of integrated care on healthcare use.

Integrated care showed a small positive effect on healthcare use for CVD and DMT2 (SMD: 0.30; 95% CI: –0.50 to 1.10) based on low-quality evidence (Table 2), with considerable heterogeneity (I2 = 57%; Figure 7). No evidence supported reduced healthcare use for CRD (Table 6 in Appendix I).

#### Secondary outcomes

##### Quality of life

**Figure 8:**
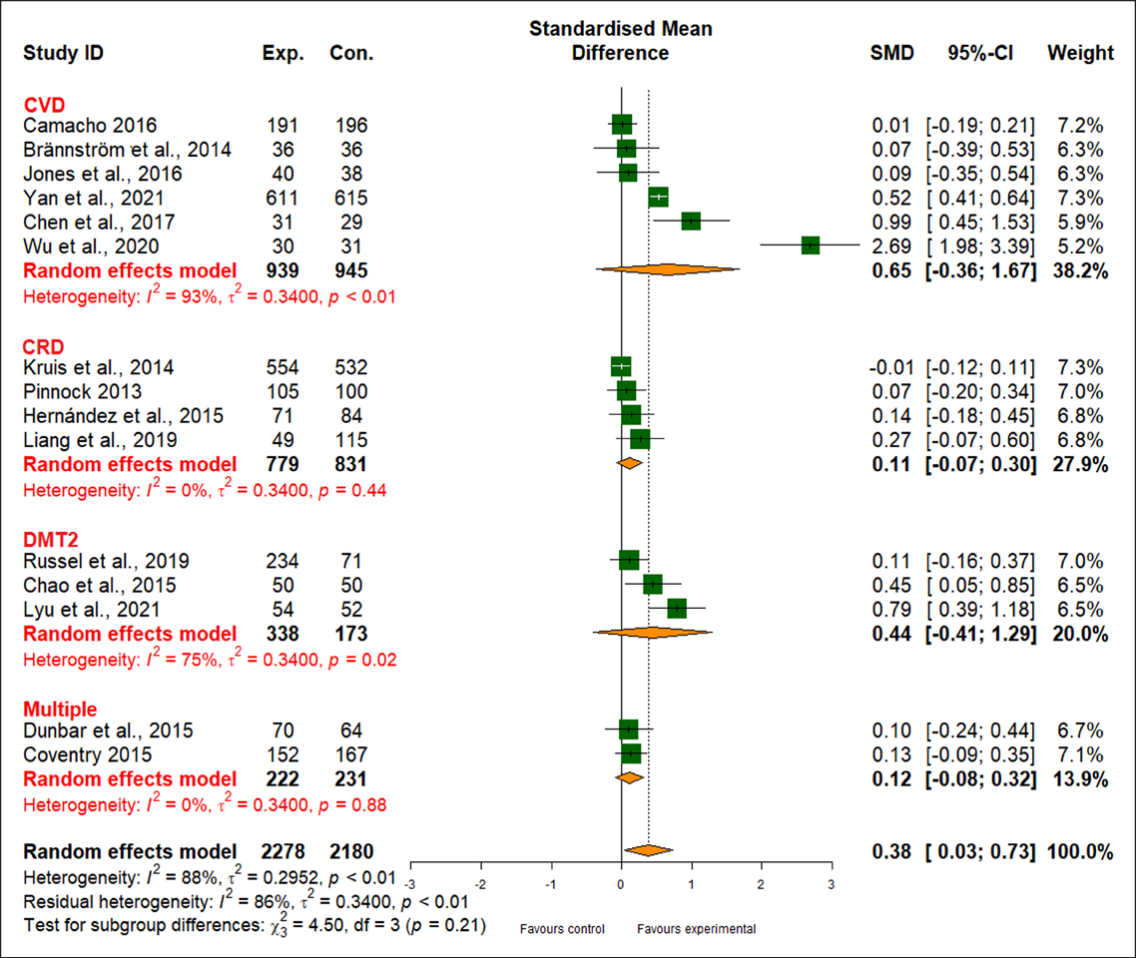
Effect of integrated care on quality of life.

Integrated care interventions positively affected health-related quality of life for CVD, DMT2, CRD, and multiple comorbidities, albeit with a high degree of variation among the reviewed studies (I2 = 88%). While different types of interventions showed some variation in effectiveness (*p*_subgroup difference_ = 0.08) (Table 6 in Appendix I), overall evidence quality was moderate (Figure 8 and Table 2). We also observed improved quality of life with integrated care for CVD and DMT2, but not for CRD or multiple comorbidities (Table 6 in Appendix I).

##### Physical functioning

**Figure 9:**
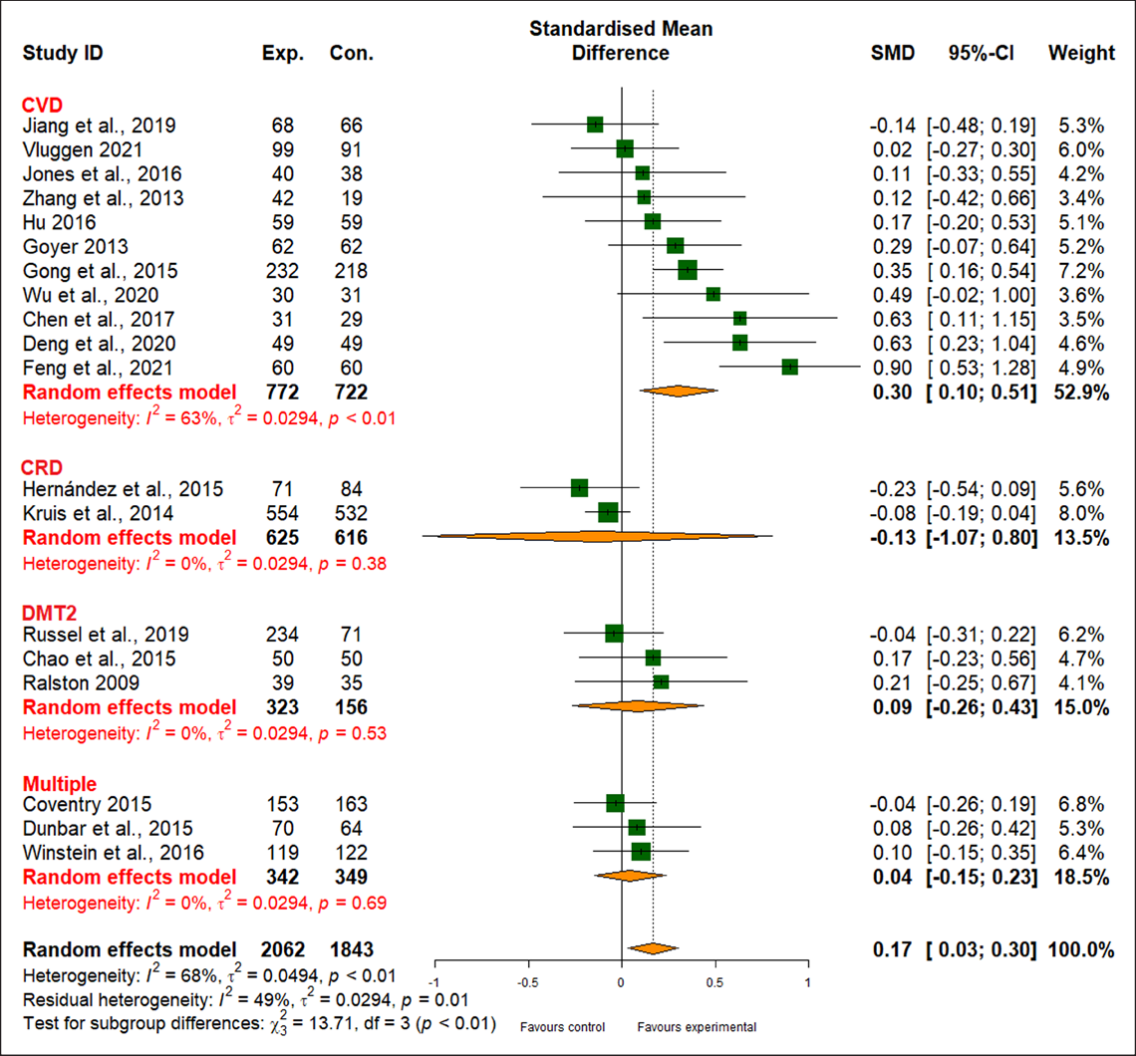
Effect of integrated care on physical functioning.

Integrated care interventions had a small positive effect on physical functioning versus standard care (SMD, 0.17; 95% CI, 0.03 to 0.30), with moderate heterogeneity (I2 = 68%). While we observed a significant improvement in physical functioning for CVD, no similar evidence was found for DMT2, CRD, or multiple comorbidities (Table 6 in Appendix I). The results also indicated a small difference in the effectiveness of different types of integrated care interventions (patient empowerment, SMD 0.20; 95% CI, 0.05 to 0.36; network care coordination, SMD 0.13; 95% CI, –0.14 to 0.40; *p*_subgroup difference_ = 0.60) (Table 6 in Appendix I). The evidence quality for functioning was moderate (Figure 9 and Table 2).

##### Weight management

**Figure 10:**
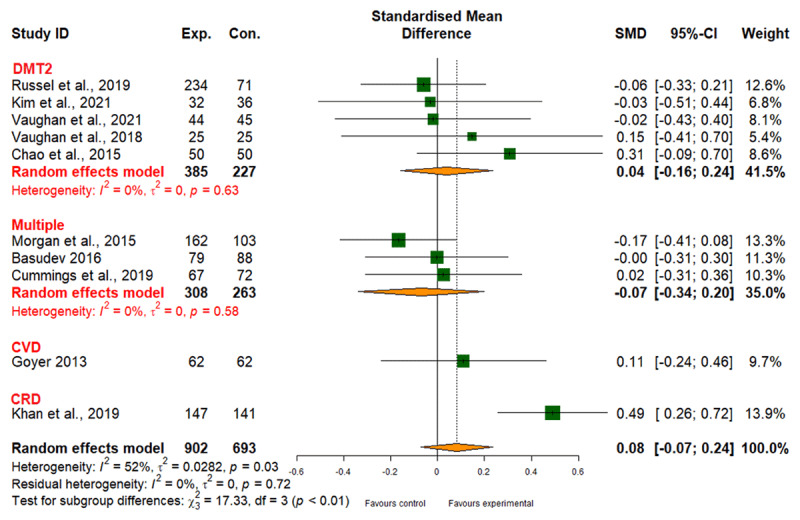
Effect of integrated care on weight management.

Integrated care had no significant impact on weight management across conditions, with moderate heterogeneity (I2 = 52%) and low evidence quality (Figure 10 and Table 2). The studies showed moderate heterogeneity (I2 = 52%) (Figure 10), with no discernible variance in the effectiveness of different integrated care interventions (patient empowerment, SMD 0.05; 95% CI, –0.09 to 0.19; network care coordination, SMD 0.10; 95% CI, –0.18 to 0.37; *p*_subgroup difference_ = 0.69) (Table 6 in Appendix I).

##### Mental health

**Figure 11:**
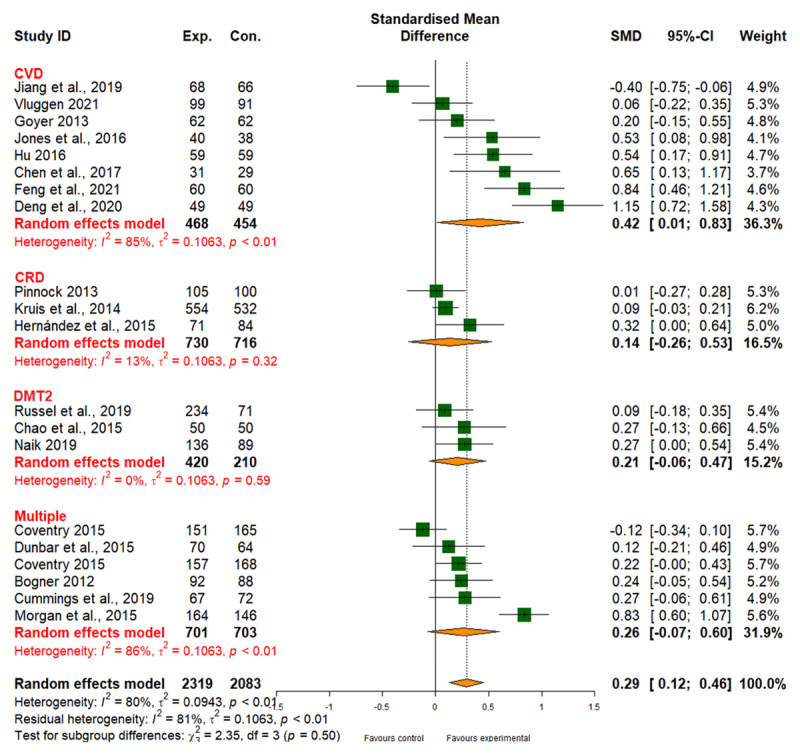
Effect of integrated care on mental health.

Integrated care positively affected mental health (SMD, 0.29; 95% CI, 0.12 to 0.46), albeit with high heterogeneity (I2 = 80%) and moderate evidence quality (Figure 11 and Table 2). No substantial evidence was obtained on different treatment effects for different types of interventions (patient empowerment, SMD 0.25; 95% CI, –0.16 to 0.66; network care coordination, SMD 0.31; 95% CI, 0.10 to 0.51; *p*_subgroup difference_ = 0.76) (Table 6 in Appendix I). with regard to individual diseases, while integrated care led to an improvement in mental health for CVD, no corresponding evidence was obtained for CRD, DMT2, or multiple comorbidities (Table 6 in Appendix I).

##### Self-management

**Figure 12:**
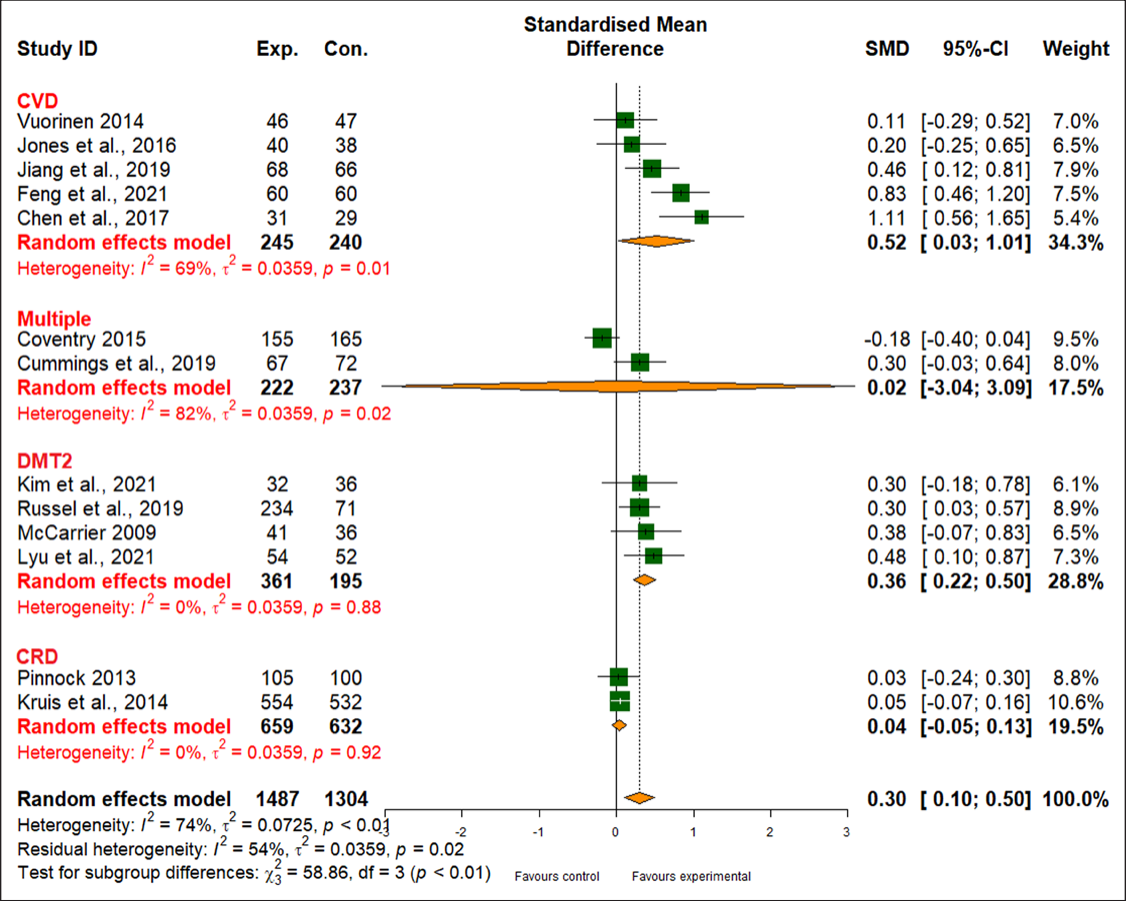
Effect of integrated care on self-management.

Compared to usual care, integrated care had a small positive effect on self-management behaviours (SMD, 0.30; 95% CI, 0.10 to 0.50), with high heterogeneity (I2 = 74%) and moderate evidence quality (Figure 12 and Table 2). No robust evidence suggested that different types of interventions affected treatment effectiveness (patient empowerment, SMD 0.49; 95% CI, 0.09 to 0.89; network care coordination, SMD 0.20; 95% CI, –0.05 to 0.45; *p*_subgroup difference_ = 0.11) (Table 6 in Appendix I). For individual diseases, integrated care had a positive effect on self-management behaviours for CVD and DMT2 (Table 6 in Appendix I), but not for CRD or multiple comorbidities.

#### Tertiary outcomes

The results on tertiary outcomes are summarized in Tables 8–10 in Appendix I and Figures 1–4 in Appendix II (cf. Table 2).

#### Qualitative synthesis

Integrated care positively affected patient knowledge and caregiver outcomes, with mixed effects on lifestyle behaviour. One study reported cost-ineffectiveness for CRD (Table 7 in Appendix I).

### Publication bias, subgroup, and sensitivity analyses

Despite limited evidence on funnel plot asymmetry for some outcomes, subgroup analyses showed no significant differences in intervention effects based on duration or study settings for most outcomes (see Tables 8–10 in Appendix I and Figures 5–17 in Appendix III for further detail).

## Discussion

### Principal findings

The key finding of our analysis of the effectiveness of integrated care interventions on health outcomes in patients with CVD, DMT2, CRD, and multiple comorbidities is that integrated care management can effectively reduce mortality, hospitalization, and adverse events rates for CVD patients. We also observed a small positive effect of integrated care interventions on the quality of life and self-management behaviour in CVD and DMT2 patients. In CVD patients, we observed a small positive impact of integrated care interventions on physical functioning and mental health. Interestingly, the effectiveness of integrated care interventions was less convincing in terms of controlling cholesterol levels, improving pulmonary function, or weight management across different disease groups. We also found limited evidence about a higher effectiveness of integrated care interventions focusing on patients, providers, and organizational levels as stipulated by the RMIC as compared to those concentrating solely on the patient-provider interface. Considering the lack of high-quality evidence on cost-effectiveness, lifestyle behaviours, knowledge, caregiver outcomes, and process-related outcomes, we cannot conclude on the effectiveness of integrated care interventions for DMT2, CVD, and CRD.

### Comparison with previous evidence

The results of this meta-analysis align with previous findings suggesting that integrated care interventions can effectively decrease mortality, hospitalization, and adverse events rates for patients with CVD [12,13,14,15]. As in previous research, we could not include studies that reported the effect of integrated care on mortality and hospitalization rates for CRD [16,88] and DMT2 [10,11,89]; hence, further research on the impact of integrated care on mortality and hospitalization rates in patients with DMT2, CRD, and multiple comorbidities is needed. Furthermore, similarly to previous research [9], the studies in our dataset did not report healthcare use data; accordingly, more research is needed on the effects of integrated care on healthcare resource use and costs in patients with chronic diseases.

We found that integrated care interventions positively affected health-related quality of life for CVD and DMT2, physical functioning for CVD, mental health for CVD, and self-management behaviour for CVD and DMT2. Importantly, our study is the first to report these findings for specifically CVD and DMT2 patients. However, unlike previous reviews [16,88], we did not find corresponding evidence for CRD. Overall, a high heterogeneity in our dataset highlights the need to meticulously design and implement integrated care interventions to ensure their effectiveness [90]. Variability in our results may also be explained by differences in target populations, study design quality, and the number of RCTs included in our analysis. Further RCT and meta-analysis are also needed to substantiate our findings on the relationship between integrated care interventions and improved quality of life, physical functioning, weight management, mental health, and self-management capabilities.

Consistently with several previous diabetes reviews [10,11,89], our results showed that integrated care interventions had a positive impact on HbA1c levels in individuals with DMT2, but not in those with CVD or multiple comorbidities. Yet, the effect of integrated care on cholesterol levels and blood pressure control remained uncertain. These findings align with previous reviews on patients with DMT2 [10,11,89]. The differences in outcomes may be explained by different study designs (i.e., pre-post studies) and type of interventions (i.e., chronic care model) included in the previous studies.

Finally, the previously reported superiority of integrated care interventions targeting the patient, provider, and organizational levels over those targeting only the patient-provider interface [5,18] remains to be substantiated in future research such as network meta-analysis [91]. One reason underlying this trend in our results can be attributed to the fact that, as in previously published integrated care reviews [17,18,58,89], relevant data on the key components of integrated care interventions were underreported in the reviewed studies.

### Strengths and limitations

To the best of our knowledge, this study is the first comprehensive analysis of various integrated care interventions for chronic care across a wide range of outcomes. Another strong aspect of the present study is that we used a robust theoretical framework, the RMIC [9], which was previously extensively used in the literature to classify and clarify ambiguous integrated care interventions.

Despite these strengths, our study has several limitations. First, the small number of studies for some of the primary outcomes (e.g., adverse events, healthcare use) included in our review may have limited the statistical power of our analyses. Second, statistical uncertainty was present in the cases of healthcare use, weight management, cholesterol, and pulmonary measurements, as the confidence interval range encompassed zero. Consequently, the observed variance between the intervention and control groups might have resulted from occurrence, rather than from a genuine distinction. For more conclusive interpretations, a larger sample size for these outcomes would be needed or more conclusive interpretations. The third limitation is that, in most of the reviewed studies, the primary outcomes had a high risk of bias, which could have adversely affected the validity of our findings. Moreover, the study setting appeared to result in significant differences in the results for some outcomes, such as adverse events and HbA1c. The risk of bias was found to be a significant factor in the results for healthcare use and blood pressure control, particularly in previous research using small sample sizes. Likewise, sample size was also found to impact the results for some outcomes, including all-cause hospital admissions, self-management, and blood pressure control. Finally, the length of follow-up was found to be a significant factor for pulmonary function, particularly in the studies focused on a small number of patients.

### Relevance for clinical practice and research

Most previous studies on integrated care for chronic diseases lack a clear definition of what constitutes integrated care. Building on the definition of integrated care proposed in the RMIC, our meta-analysis underscores the need for primary research to identify which specific combination of integrated care interventions can enhance outcomes for patients with chronic diseases. Since most of the previous studies focused on individual integrated care interventions targeting the patient-provider interface, our results highlight that future research should focus on improving the longitudinal design and evaluating different types of interventions. Considering that integrated care interventions become increasingly prevalent, our findings should also spur interest in implementing real-world evaluation designs of innovative care models to improve health care delivery. Since none of the reviewed studies concurrently addressed the triple aim of evaluating health, quality of care, and cost outcomes, our meta-analysis underscores the importance of developing a core set of triple-aim outcomes for integrated care interventions including a well-defined set of measures evaluating user experience, intervention quality, and costs for individuals with CVD, DMT2, CRD, and multiple comorbidities.

## Conclusions

The results of our meta-analysis suggest that integrated care interventions targeting patients with chronic diseases can have moderate-quality positive effects on all-cause mortality, reducing adverse events, health-related quality of life, physical functioning, mental health, self-management behaviours, and blood pressure control improvement. However, the evidence for some primary outcomes, such as all-cause hospital admissions and healthcare use, remains inconclusive due to the small number of studies for some of the outcomes. This warrants further research that would use longitudinal designs, focus on different types of interventions, and draw on clear evidence-based definitions of integrated care, to better evaluate the triple aim of improving health, quality of care, and cost outcomes for patients with chronic diseases.

## Additional File

The additional file for this article can be found as follows:

10.5334/ijic.7744.s1 Appendices. Appendix I to III.
