# Supplementary material for: Effectiveness of Integrated Care for Diabetes Mellitus Type 2, Cardiovascular and Chronic Respiratory Diseases: A Systematic Review and Meta-Analysis
Source: Int J Integr Care. 2024 Aug 19;24(3):16. doi: 10.5334/ijic.7744 (PMC11342834; doi:10.5334/ijic.7744)
Supplement: Appendices. — Appendix I to III. [file ijic-24-3-7744-s1.pdf]

Appendices

Appendix 1

Table 1 Search strategy.

	Database	Date	Keywords	Results	Link
1	PubMed	7-9-2021	(((((Integrated[Title]) OR (Interdisciplinary[Title])) OR (Collaborative[Title])) OR (Multidisciplinary[Title])) AND (Diabetes[Title])) AND (Randomized[Title])	59	Link
			(((((Integrated[Title]) OR (Interdisciplinary[Title])) OR (Collaborative[Title])) OR (Multidisciplinary[Title])) AND (Diabetic[Title])) AND (Randomized[Title])	1	Link
			(((((Integrated[Title]) OR (Interdisciplinary[Title])) OR (Collaborative[Title])) OR (Multidisciplinary[Title])) AND (Heart[Title])) AND (Randomized[Title])	45	Link
			(((((Integrated[Title]) OR (Interdisciplinary[Title])) OR (Collaborative[Title])) OR (Multidisciplinary[Title])) AND (cardiovascular[Title])) AND (Randomized[Title])	20	Link

			(((((Integrated[Title]) OR (Interdisciplinary[Title])) OR (Collaborative[Title])) OR (Multidisciplinary[Title])) AND (COPD[Title])) AND (Randomized[Title])	2	Link
			(((((Integrated[Title]) OR (Interdisciplinary[Title])) OR (Collaborative[Title])) OR (Multidisciplinary[Title])) AND (Chronic Obstructive Pulmonary Disease[Title])) AND (Randomized[Title])	2	Link
2	Google Scholar	7-9-2021	allintitle: Integrated randomized Diabetes OR DM OR Diabetic OR Noninsulin OR T2DM	32	Link
			allintitle: Collaborative randomized Diabetes OR DM OR Diabetic OR Noninsulin OR T2DM	26	Link
			allintitle: Multidisciplinary randomized Diabetes OR DM OR Diabetic OR Noninsulin OR T2DM	9	Link
			allintitle: Interdisciplinary randomized Diabetes OR DM OR Diabetic OR Noninsulin OR T2DM	5	Link
			allintitle: Integrated randomized Cardiovascular OR heart OR Stroke OR Ischemic	50	Link
			allintitle: Collaborative randomized Cardiovascular OR heart OR Stroke OR Ischemic	55	Link

			allintitle: Multidisciplinary randomized Cardiovascular OR heart OR Stroke OR Ischemic	23	Link
			allintitle: Interdisciplinary randomized Cardiovascular OR heart OR Stroke OR Ischemic	10	Link
			allintitle: bronchial Asthma Randomized Collaborative OR Integrated OR Interdisciplinary OR Multidisciplinary	0	Link
			allintitle: COPD Randomized Collaborative OR Integrated OR Interdisciplinary OR Multidisciplinary	8	Link
			allintitle: chronic obstructive pulmonary disease Randomized Collaborative OR Integrated OR Interdisciplinary OR Multidisciplinary	7	Link
3	Web of Science	7-9-2021	**Integrated** (Title)or **Collaborative** (Title) or **Multidisciplinary** (Title) and **Diabetes** (Title) or **Diabetic** (Title) and **Randomized** (Title) and **2011** or **2012** or **2013** or **2014** or **2015** or **2016** or **2018** or **2017** or **2019** or **2020** or **2021** (Publication Years)	41	Link
			**Integrated** (Title)or **Collaborative** (Title) or **Multidisciplinary** (Title) and **Heart** (Title) or **Stroke** (Title) and **Ischemic Heart Failure** (Title) and **Randomized** (Title)and **2011** or **2012** or **2013** or **2014** or **2015** or **2016** or **2018** or **2017** or **2019** or **2020** or **2021** (Publication Years)	40	Link

			(TITLE (integrated) OR TITLE (collaborative) OR TITLE (multidisciplinary) OR TITLE (interdisciplinary) AND TITLE (diabetes AND mellitus) OR TITLE (diabetic) OR TITLE (dm) OR TITLE (t2dm) AND TITLE (randomized))	13	Link
4	Scopus	7-9-2021	(TITLE (integrated) OR TITLE (collaborative) OR TITLE (multidisciplinary) OR TITLE (interdisciplinary) AND TITLE (heart) OR TITLE (cardiovascular) OR TITLE (ischemic AND heart AND disease) OR TITLE (stroke) AND TITLE (randomized)) AND (LIMIT-TO (PUBYEAR , 2021) OR LIMIT-TO (PUBYEAR , 2020) OR LIMIT-TO (PUBYEAR , 2019) OR LIMIT-TO (PUBYEAR , 2018) OR LIMIT-TO (PUBYEAR , 2017) OR LIMIT-TO (PUBYEAR , 2016) OR LIMIT-TO (PUBYEAR , 2015) OR LIMIT-TO (PUBYEAR , 2014) OR LIMIT-TO (PUBYEAR , 2013) OR LIMIT-TO (PUBYEAR , 2012) OR LIMIT-TO (PUBYEAR , 2011))	92	Link
			(TITLE (integrated) OR TITLE (collaborative) OR TITLE (multidisciplinary) OR TITLE (interdisciplinary) AND TITLE (copd) OR TITLE (bronchial AND asthma) OR TITLE (chronic AND obstructive AND pulmonary AND disease) AND TITLE (randomized)) AND (LIMIT-TO (PUBYEAR , 2021) OR LIMIT-TO (PUBYEAR , 2020) OR LIMIT-TO (PUBYEAR , 2019) OR LIMIT-TO (PUBYEAR , 2018) OR LIMIT-TO (PUBYEAR , 2017) OR LIMIT-TO (PUBYEAR , 2016) OR LIMIT-TO (PUBYEAR , 2015) OR LIMIT-TO (PUBYEAR , 2014) OR LIMIT-TO (PUBYEAR , 2013))	16	Link
5	EMBASE	7-9-2021	(integrated:ti OR coordinated:ti OR collaborative:ti OR multidisciplinary:ti OR interdisciplinary:ti) AND 'diabetes mellitus':ti AND randomized:ti	11	Link
			(integrated:ti OR coordinated:ti OR collaborative:ti OR multidisciplinary:ti OR	2	Link

			interdisciplinary:ti) AND 'diabetic':ti AND randomized:ti		
			(integrated:ti OR coordinated:ti OR collaborative:ti OR multidisciplinary:ti OR interdisciplinary:ti) AND 'heart':ti AND randomized:ti	44	Link
			(integrated:ti OR coordinated:ti OR collaborative:ti OR multidisciplinary:ti OR interdisciplinary:ti) AND 'cardiovascular':ti AND randomized:ti	19	Link
			(integrated:ti OR coordinated:ti OR collaborative:ti OR multidisciplinary:ti OR interdisciplinary:ti) AND 'copd':ti AND randomized:ti	10	Link
			(integrated:ti OR coordinated:ti OR collaborative:ti OR multidisciplinary:ti OR interdisciplinary:ti) AND 'chronic obstructive pulmonary disease':ti AND randomized:ti	4	Link
			"Integrated" OR " Collaborative" AND" Diabetes mellitus" OR" Diabetic" OR "DM" AND" Randomized"	0	Link
6	SIGLE	7-9-2021	"Integrated" AND" Heart" OR" Stroke" OR "Ischemic Heart Disease" AND" Randomized"	0	Link
			"Integrated" AND" COPD" OR" Bronchial Asthma" OR "Chronic Obstructive Pulmonary Disease" AND" Randomized"	0	Link

			(ti:(Integrated)) OR (ti:(Cooperative)) OR (ti:(collaborative)) OR (ti:(Multidisciplinary)) OR (ti:(Interdisciplinary)) AND (ti:(Diabetes Mellitus)) OR (ti:(Diabetic)) AND (ti:(Randomized))	1	Link
7	Virtual Health Library	7-9-2021	(ti:(Integrated)) OR (ti:(Cooperative)) OR (ti:(Collaborative)) OR (ti:(Interdisciplinary)) OR (ti:(Multidisciplinary)) AND (ti:(Heart)) OR (ti:(Stroke)) OR (ti:(Cardiovascular)) AND (ti:(Randomized))	2	Link
			(ti:(Integrated)) OR (ti:(Cooperative)) OR (ti:(collaborative)) OR (ti:(Multidisciplinary)) OR (ti:(Interdisciplinary)) AND (ti:(COPD)) OR (ti:(Chronic Obstructive Pulmonary Disease) OR (ti:(Bronchial Asthma) AND (ti:(Randomized))	0	Link
			"Integrated" OR " Collaborative" AND" Diabetes mellitus" OR" Diabetic" OR "DM" AND" Randomized"	0	Link
8	NYAM	7-9-2021	"Integrated" AND" Heart" OR" Stroke" OR "Ischemic Heart Disease" AND" Randomized"	0	Link
			"Integrated" AND" COPD" OR" Bronchial Asthma" OR "Chronic Obstructive Pulmonary Disease" AND" Randomized"	0	Link

9	Clinical Trials.Gov	7-9-2021	Studies With Results | Diabetes | integrated CARE	2	Link
			Studies With Results | Heart | integrated CARE	3	Link
			Studies With Results | Stroke | integrated CARE	2	Link
			Studies With Results | Chronic Obstructive Pulmonary Disease | integrated CARE	2	Link
			Studies With Results | Bronchial Asthma | integrated CARE	2	Link
10	Controlled Trials (mRCT)	7-9-2021	Condition: Diabetes Interventions: Integrated Care	2	Link
			Condition: Heart Interventions: Integrated Care	1	Link
			Condition: Stroke Interventions: Integrated Care	2	Link

11	Cochrane Central Register of Controlled Trials	7-9-2021	Integrated in Record Title OR Multidisciplinary in Record Title OR "collaborative" in Record Title AND Diabetes in Record Title - (Word variations have been searched	25	Link
			Integrated in Record Title OR Multidisciplinary in Record Title OR "collaborative" in Record Title AND Heart in Record Title - (Word variations have been searched)	25	Link
			Integrated in Record Title OR Multidisciplinary in Record Title OR "collaborative" in Record Title AND Stroke in Record Title - (Word variations have been searched)	25	Link

Table 2 Description of the integrated health interventions.

Domain	Intervention	Description
Organizational level	Disease management	A system of coordinated healthcare interventions and communications for populations with conditions in which patient self-care efforts are significant. Disease management supports the physician or practitioner/patient relationship and plan of care, emphasizes prevention of exacerbations and complications utilizing evidence-based practice guidelines and patient empowerment strategies, and evaluates clinical, humanistic, and economic outcomes on an ongoing basis with the goal of improving overall health. It includes: 1) population identification process, 2) evidence-based practice guidelines, 3) collaborative practice model, 4) self-management education for patients, 5) process and outcomes measurement, 6) routine reporting, feedback loops and benchmarking.
	Managed care programs	Health insurance plans intended to reduce unnecessary health care costs through a variety of mechanisms, including: economic incentives for physicians and patients to select less costly forms of care; programs for reviewing the medical necessity of specific services; increased beneficiary cost sharing; controls on inpatient admissions and lengths of stay; the establishment of cost-sharing incentives for outpatient surgery; selective contracting with health care providers; and the intensive management of high-cost health care cases. The programs may be provided in a variety of organisational settings, such as health maintenance organizations (HMO’s), preferred provider organizations (PPOs), integrated delivery system (IDS) or accountable care organizations (ACOs).

	Electronic Patient Registry	General electronic medical record system or electronic tracking system for patients which is shared across different care providers.
	Clinical information transmission	Clinical information collected from patients and transmitted to clinicians by means other than the existing medical record. Examples include elector or web-based methods through which patients provided self-care data and which clinics reviewed. In general, the patient should be facilitating the relay.
Professional domain	Multidisciplinary team	Interventions comprising teams composed of multiple health and/or social care professionals working together to provide care for people with complex needs. Teams typically included condition-specific expertise, nurses, occupational therapists, physiotherapists, social workers, GPs and occasionally pharmacists or case managers.

Continuity of care	The degree to which a series of discrete healthcare events is experienced as coherent and connected and consistent with the patient's medical needs and personal context. It includes (1) Informational continuity: The use of information on past events and personal circumstances to make current care appropriate for each individual. (2) Management continuity: A consistent and coherent approach to the management of a health condition that is responsive to a patient's changing needs. (3) Relational continuity: An ongoing therapeutic relationship between a patient and one or more providers.
Clinician education	Interventions designed to promote increased understanding of principles guiding clinical care or awareness of specific recommendations for a target condition or patient population. Subcategories of clinician education included conferences or workshops, distribution of educational materials, and educational outreach visits.
Audit and feedback	Summary of clinical performance of health care delivered by an individual clinician or clinic over a specified period, which is then transmitted back to the clinician (e.g., the percentage of a clinician’s patients who have achieved a target glycosylated hemoglobin [HbA1c] level, or who have undergone a dilated-eye examination with a specified frequency).
Clinician Reminders	Paper-based or electronic system intended to prompt a health professional to recall patient-specific information (e.g., most recent HbA1c value) or to perform a specific task (e.g., perform a foot examination). If accompanied by a recommendation, the strategy would be sub-classified as decision support.

Patient domain	Case management	Based on implementation of a collaborative process between one or more care coordinators or case managers and the patient, to assess, plan and facilitate service delivery for patients with chronic diseases, particularly when transitions across healthcare settings are required.
	Integrated care pathway	Structured multidisciplinary care plans which detail essential steps in the care of individual patients with a specific clinical problem and describe the patient's expected clinical course.
	Discharge management	Interventions designed to facilitate effective transitions from hospital care to other settings. Typically includes a pre-discharge phase of support, transitional care for the move between the hospital and community/home setting and post-discharge follow-up and monitoring, often incorporating rehabilitation or reablement support.
	Self-management	Interventions designed to provide patient support, typically via tailored education to inform the patient about their condition(s), recognising signs and symptoms of disease exacerbation, dietary and lifestyle advice and/or condition-specific education supporting medication adherence.<br><br>Interventions designed to promote increased understanding of a target condition or to teach specific prevention or treatment strategies, or specific in-person patient education (e.g., individual or group sessions with diabetes nurse educator; distribution of printed or electronic educational materials).

	Any effort (e.g., postcards or telephone calls) to remind patients about upcoming appointments or important aspects of self-care.
Patient education	
Patient reminders	

Table 3 Description of studies included in the systematic review.

Study	Country	Target population	Setting	Intervention level (RMIC)	Intervention type (WHO)	Outcome assessed
[23]	Brazil	Type 2 diabetes	Clinic	1. Patient domain<br>2. Professional domain<br>3. Organizational domain	- Self-management<br>- Integrated care pathway<br>- Clinician education<br>- Electronic patient registry<br>- Clinical information transmission	1. Number of medications<br>2. Medication Regimen Complexity Index<br>3. Number of anti-diabetic drugs<br>4. Medication adherence<br>5. Adherence score<br>6. HbA1c<br>7. Systolic blood pressure<br>8. Diastolic blood pressure<br>9. LDL- cholesterol
[24]	India	Type 2 diabetes,<br>depression, at least<br>1 poorly controlled	Clinic	1. Patient domain<br>2. Professional domain<br>3. Organizational domain	- Case management<br>- Integrated care pathway<br>- Patient education	1. PHQ-9[a]<br>2. Systolic blood pressure<br>3. Hb1Ac

		cardiometabolic parameter			- Multidisciplinary team<br>- Clinician education<br>- Audit and feedback<br>- Disease management<br>- Electronic patient registry	4. SCL-20[b]<br>5. HDL- cholesterol<br>6. LDL- cholesterol<br>7. Total cholesterol<br>8. Fasting blood glucose
[25]	South London	Type 2 diabetes, depression	GP clinic	1. Patient domain<br>2. Professional domain<br>3. Organizational domain	- Case management<br>- Integrated care pathway<br>- Self-management<br>- Multidisciplinary team<br>- Continuity of care<br>- Clinician education<br>- Disease management<br>- Clinical information transmission	1. HbA1c<br>2. Lipids<br>3. Blood pressure<br>4. Weight (kg and BMI)<br>5. Renal function (eGFR)
[26]	USA	Type 2 diabetes	Urban	1. Patient domain	- Case management	1. Readmission rates

			teaching hospital	2. Professional domain	- Integrated care pathway	2. HbA1c
					- Discharge management	
					- Multidisciplinary team	
					- Continuity of care	
[27]	USA	Type 2 diabetes,	Primary	1. Patient domain	- Case management	1. MMSE[c]
		depression	care	2. Professional domain	- Integrated care pathway	2. HbA1c
			facility	3. Organizational domain	- Patient education	3. PHQ-9[a]
					- Multidisciplinary team	4. SF-36[d]
					- Continuity of care	5. BMI[e]
					- Clinician education	6. LDL - cholesterol
					- Disease management	
[28]	Netherlands	COPD	Primary	1. Patient domain	- Self-management	1. EQ-5D[f]
			care	2. Professional domain	- Multidisciplinary team	2. Clinical COPD Questionnaire
			teams	3. Organizational domain	- Clinician education	3. St. George’s Respiratory Questionnaire

					- Audit and feedback	4. Total number of COPD exacerbations
					- Managed care programs	5. Costs
[29]	Germany	Cardiovascular	Clinic	1. Patient domain	- Case management	1. Blood pressure
		disease (CVD)		2. Professional domain	- Integrated care pathway	2. LDL - cholesterol
				3. Organizational domain	- Patient education	3. HbA1c
					- Multidisciplinary team	4. Self-reports of tobacco use
					- Clinician education	5. PSS-4[g]
					- Audit and feedback	6. HADS[h]
					- Disease management	7. HAF-17[i]
					- Electronic patient registry	8. HRQoL[j]
						9. SF-12; mental (MCS)[d]
						10. SF-12 physical components (PCS)[d]
						11. GSE-6[k]
						12. ESSI[l]

[30]	Sweden	Patients with diagnosed coronary heart disease	Hospital / home	1. Patient domain<br>2. Professional domain<br>3. Organizational domain	- Case management<br>- Self-management<br>- Multidisciplinary team<br>- Continuity of care<br>- Disease management	1. EQ-5D[f]<br>2. ESAS[m]<br>3. KCCQ[n]<br>4. Functional classes<br>5. Hospitalizations<br>6. Resource utilization
[31]	England	Cardiovascular disease (CVD)	Primary care facility	1. Patient domain<br>2. Professional domain<br>3. Organizational domain	- Case management<br>- Integrated care pathway<br>- Discharge management<br>- Multidisciplinary team<br>- Continuity of care<br>- Disease management<br>- Managed care programs	1. PHQ-9[a]<br>2. SCL-D13<br>3. EQ -5D-5L[f]<br>4. EQ-5D[f]<br>5. WHO-QOL[o]<br>6. SCL-90[b]<br>7. GAD-7[p]<br>8. Symptom Disruption

						Score (SDS)
[32]	Italy	Adult obese patients with type-2 diabetes mellitus	Hospital / home	1. Patient domain<br>2. Professional domain<br>3. Organizational domain	- Discharge management<br>- Patient education<br>- Patient reminders<br>- Continuity of care<br>- Clinical Education<br>- Clinician Reminders<br>- Managed care programs<br>- Clinical information transmission	1. Weight<br>2. Eating Disorder Inventory (EDI-2)
[33]	China	Elderly (+60) diagnosed with type 2 diabetes	Hospital	1. Patient domain<br>2. Professional domain 3. Organizational domain	- Integrated care pathway<br>- Patient education<br>- Case management<br>- Multidisciplinary team<br>- Continuity of care	1. Health knowledge score<br>2. Self-evaluated psychological health status score<br>3. Self-evaluated health status score<br>4. Diet<br>5. Physical activity duration

					- Disease management	6. BMI<br>7. Waist-to-hip ratio (WHR)<br>8. Systolic Blood pressure<br>9. Diastolic blood pressure<br>10. Fasting blood glucose<br>11. Number of outpatient clinic visits<br>12. Number of admissions in the preceding<br>13. The days of hospital admissions
[34]	China	Patients with heart failure diagnosis and<br>NYHA class II to IV	Hospital /<br>home	1. Patient domain<br>2. Professional domain	- Discharge management<br>- Multidisciplinary team<br>- Clinician education	1. Minnesota Living with Heart Failure Questionnaire<br>2. Short Physical Performance Battery<br>3. PHQ-9[a]<br>4. European Heart Failure Self-care Behaviour Scale
[35]	Spain	Heart failure	Clinic/	1. Patient domain	- Integrated care pathway	1. non-fatal HF events

			home	2. Professional domain	- Discharge management	2. All-cause and HF Hospitalization
				3. Organizational domain	- Self-management	3. All-cause and CV death
					- Multidisciplinary team	4. Psychosocial Self-efficacy points
					- Continuity of care	4. Blood pressure (BP)
					- Disease management	5. BMI[e]
					- Clinical information transmission	6. Heart rate
						7. left ventricular ejection fraction (LVEF)
						8. Hemoglobin
						9. eGFR
[36]	England	Type 2 diabetes, depression	GP clinic	1. Patient domain	- Case management	1. SCL-D13[b]
				2. Professional domain	- Integrated care pathway	2. PHQ-9[a]
				3. Organizational domain	- Self-management	3. GAD-7[p]
					- Multidisciplinary team	4. WHO-QOL-BREF[o]
					- Clinician education	5. PACIC[Q]

					- Audit and feedback	6. Stroke Self-Efficacy Questionnaire (SSEQ)
					- Disease management	7. ESSI[l]
					- Electronic patient registry	8. Client Satisfaction Questionnaire (CSQ)
[37]	Canada	Chronic and severe heart failure	Clinic	1. Patient domain	- Case management	1. HbA1c
				2. Professional domain	- Self-management	2. Systolic blood pressure
				3. Organizational domain	- Patient education	3. Weight
					- Multidisciplinary team	4. Regimen-related distress score
					- Continuity of care	5. PHQ-9[a]
					- Disease management	6. Self-care activities score
						7. Medication adherence score
[38]	China	Type 2 diabetic patients	Hospital / community health centre	1. Patient domain	- Self-management	1. SF-36, Physical Components
				2. Professional domain	- Discharge management	2. SF-36, Mental Components
				3. Organizational domain	- Case management	3. Modified Barthel Index
					- Multidisciplinary team	4. Caregiver Strain Index

					- Disease management	
[39]	New Zealand	Patients with chronic heart failure	Hospital	1. Patient domain<br>2. Professional domain<br>3. Organizational domain	- Case management<br>- Integrated care pathway<br>- Discharge management<br>- Multidisciplinary team<br>- Continuity of care<br>- Disease management<br>- Electronic patient registry	1. Death<br>2. Hospital readmission<br>3. Time to the first hospital readmission<br>4. Quality of life questionnaire
[40]	Canada	Cardiovascular disease (CVD)	Hospital	1. Patient domain<br>2. Professional domain<br>3. Organizational domain	- Case management<br>- Integrated care pathway<br>- Discharge management<br>- Multidisciplinary team<br>- Continuity of care<br>- Audit and feedback	1. all-cause hospital admission rates<br>2. total number of days in hospital<br>3. total number of emergency visits<br>4. quality of life (Minnesota QoL)<br>5. total mortality

					- Disease management<br>- Clinical information transmission	
[41]	USA	Patients with current or recent hospitalization for heart failure within the past three months and diabetes type 2	Hospital/ home	1. Patient domain	- Self-management<br>- Patient education	1. Minnesota Living with Heart Failure<br>2. Minnesota Living with Heart Failure physical<br>3. Minnesota Living with Heart Failure emotional<br>4. Audit of Diabetes-Dependent Quality of Life<br>5. EuroQol 5 Dimension<br>6. EuroQol Visual Analogue Scale
[42]	USA	Low income with diabetes and depression	Clinic/ home/ hospital	1. Patient domain<br>2. Professional domain<br>3. Organizational domain	- Case management<br>- Discharge management<br>- Patient education<br>- Multidisciplinary team<br>- Continuity of care<br>- Disease management	1. PHQ-9[a]<br>2. SCL-90[b]<br>3. Structured Clinical Interview for DSM (SCID)<br>4. Whitty 9-item questionnaire<br>Diabetes Self-Care Activities Questionnaire<br>5. Brief Symptom Inventory (BSI) anxiety

					- Clinical information transmission	module<br>6. SF-12d<br>7. Severity of Dependence Scale (SDS)<br>8. HbA1c
[43]	China	Heart failure patients<br>having functional<br>class III/IV CHF and<br>judged to be<br>at high risk of<br>CHF readmission	Hospital / community health<br>centre	1. Patient domain<br>2. Professional domain<br>3. Organizational domain	- Discharge management<br>- Self-management<br>- Case management<br>- Multidisciplinary team<br>- Continuity of care<br>- Electronic patient registry	1. Modified Barthel Index<br>2. Medication according to doctor's advice<br>3. Reasonable diet<br>4. Moderate exercise<br>5. Regular follow-up visits<br>6. General self-efficacy scale 7. Zung's Self-rating Anxiety Scale<br>8. Zung's Self-rating Depression Scale
[44]	China	Elderly 55 year or older diagnosed with<br>hypertension by<br>a doctor or a physician	Community / home	1. Patient domain<br>2. Professional domain<br>3. Organizational domain	- Patient education<br>- Clinician education<br>- Disease management	1. Tendency toward physically active<br>2. Levels of physical activity 3. Systolic Blood pressure<br>4. Diastolic blood pressure

						5. Incidence rate (%) of heart attack<br>6. Incidence rate (%) of stroke
[45]	Canada	Cardiovascular disease (CVD)	Clinic	1. Patient domain<br>2. Professional domain<br>3. Organizational domain	- Self-management<br>- Patient education<br>- Multidisciplinary team<br>- Disease management	1. Cardiovascular risk (Framingham and procam)<br>2. BMI[e]<br>3. Blood pressure (SBP & DBP)<br>4. Cholesterol<br>5. HbA1c<br>6. SF-12/MCS[d]<br>7. Beck Depression Inventory – Second Edition (BDI-II)<br>8. VO2-max<br>9. Weight<br>10. Waist circumference (cm)<br>11. HDL-C (mmol/l)

						12. LDL-C (mmol/l)
						13. Triglycerides
						14. Glucose (mmol/l)
						15. Physical activity (mets*h/week)
						16. Energy (kcal)
[46]	Netherland	Atrial fibrillation	Hospital/	1. Patient domain	- Case management	1. Cardiovascular hospitalization
			clinic	2. Professional domain	- Integrated care pathway	2. cardiovascular death
				3. Organizational domain	- Self-management	3. Heart rate
					- Multidisciplinary team	4. Left ventricular ejection fraction (LVEF)
					- Continuity of care	5. Blood pressure
					- Clinician education	6. BMI[e]
					- Disease management	
					- Electronic patient registry	

[47]	Spain	Patients with chronic heart failure	Clinic / home / hospital	1. Patient domain<br>2. Professional domain<br>3. Organizational domain	- Case management<br>- Multidisciplinary team<br>- Clinician education<br>- Electronic patient registry	1. Active smokers<br>2. MRC dyspnoea scale<br>3. Lawton index<br>4. Hospital Anxiety scale<br>5. Hospital Depression scale 6. Quality of life (SGRQ)<br>7. Influenza vaccination<br>8. Pneumococcal vaccination<br>9. LTOT<br>10. Long-acting B-agonists 11. Anticholinergics<br>12. Inhaled steroids<br>13. COPD knowledge and self-management
[48]	China	Heart failure	Hospital/ home	1. Patient domain<br>2. Professional domain	- Self-management<br>- Patient education<br>- Multidisciplinary team	1. SF-36 (PCS and MCS)[d]<br>2. Zarit Burden Interview (ZBI)<br>3. CES-D[r]

[49]	USA	Type 2 diabetic patients	Advantage Health Physician Network	1. Patient domain<br>2. Professional domain	- Integrated care pathway<br>- Self-management<br>- Patient education<br>- Multidisciplinary team<br>- Continuity of care	1. Reduction in HbA1c %
[50]	China	Patients with diagnosed xoronary heart disease	Health centre	1. Patient domain<br>2. Professional domain	- Case management<br>- Self-management<br>- Patient education<br>- Multidisciplinary team<br>- Clinician education<br>- Continuity of care	1. Coronary Artery Disease Self-Management Scale<br>2. Self-efficacy for Chronic Disease 6-item Scale Confidence in Controlling Symptom;<br>3. Self-efficacy for Chronic Disease 6-item Scale Confidence in Maintaining Function<br>4. Short Form-12 item health survey questionnaire physical<br>5. Short Form-12 item health survey questionnaire mental
[51]	Spain	Heart failure	Home-base	1. Patient domain<br>2. Professional domain	Case management<br>- Discharge management	1. Incidence of acute HF<br>2. Healthcare-related costs

				3. Organizational domain	- Patient education	3. LVEF
					- Multidisciplinary team	4. self-efficacy points
					- Audit and feedback	5. BP
					-Clinician reminders	6. BMI[e]
					- Disease management	7. Heart rate
					- Electronic patient registry	8. Hb
						9. eGFR
[52]	UK	Patients with stroke	Community / home	1. Patient domain	-Self-management	1. Nottingham Extended Activities of Daily Living Scale
		referred for CSR		2. Professional domain	- Multidisciplinary team	2. Hospital Anxiety and Depression Scale — Anxiety scores;
					- Clinician education	3. Hospital Anxiety and Depression Scale — Depression scores
						4. Stroke and Aphasia Quality of Life scores
						5. Short Form 12 questionnaire- physical;
						6. Short Form 12 questionnaire- mental

						7. Stroke Self-Efficacy Questionnaire
[53]	USA	Heart failure patients having functional class III/IV CHF and judged to be at high risk of CHF readmission	Clinic/ home/ hospital	1. Patient domain<br>2. Professional domain<br>3. Organizational domain	- Case management<br>- Self-management<br>- Multidisciplinary team<br>- Continuity of care<br>- Clinician reminders<br>- Disease management<br>- Electronic patient registry	1. Death<br>2. CHF hospital admissions<br>3. All-cause hospital admissions<br>4. Minnesota score<br>5. Duke Activity Status<br>costs per patient<br>6. BP<br>7. pulse
[54]	Pakistan	Patients newly diagnosed with COPD	Health facilities	1. Patient domain<br>2. Professional domain<br>3. Organizational domain	- Case management<br>- Self management<br>- Multidisciplinary team<br>- Disease management<br>- Managed care programs	1. BODE index score change 2. COPD control<br>3. Quit rate among smokers 4. Follow-up adherence

[55]	South Korea	Adults diagnosed with diabetes type 2, taking diabetes or insulin medication for more than 6 months	Hospital / home	1. Patient domain<br>2. Professional domain<br>3. Organizational domain	- Self-management<br>- Patient education<br>- Clinician education<br>- Clinician reminders<br>- Disease management	1. HbA1c (%)<br>2. BMI (kg/m2)<br>3. Fasting blood sugar FBS 4. Daily steps count (step/day) 5. Total calorie intake (kcal/day) 6. Carbohydrate intake (g/day) 7. Protein intake (g/day) 8. Fat intake (g/day) 9. The Brief Diabetes Knowledge Test (Knowledge) 10. the third version of the Diabetes Attitude Scale (DAS-3) (Personal motivation) 11. the Diabetes Family Behaviour Checklist-2 (Social motivation) 12. the Diabetes Self-Efficacy Scale (Behavior skills) 13. the Revised Summary of Diabetes Self-Care Activities Measure Scale (Self-management Behaviours)
[56]	Netherlands	Patients diagnosed with COPD according to GOLD guidelines	GP clinic	1. Patient domain<br>2. Professional domain<br>3. Organizational domain	-Case management<br>- Multidisciplinary team<br>-Audit and feedback<br>- Clinical education<br>- Disease management<br>- Managed care programs	1. Clinical COPD Questionnaire<br>2. St George's Respiratory Questionnaire<br>3. Medical Research Council<br>4. Euro Qol-5D<br>6. Short Form 36<br>7. Self-Management Ability Scale<br>8. International Physical Activity Questionnaire<br>9. Patient Assessment Chronic Illness Care

						10. Smokers rate<br>11. Hospital admission days<br>12. Moderate exacerbations<br>13. Severe exacerbations
[57]	Germany	Type 2 Diabetes patients	Health facilities	1. Patient domain<br>2. Professional domain<br>3. Organizational domain	- Case management<br>- Self-management<br>- Patient education<br>- Continuity of care<br>- Clinician education<br>- Disease management<br>- Electronic patient registry	1. Glycemic control (HbA1c)<br>2. SMBG<br>3. Hypoglycemic episodes<br>4. Blood pressure<br>5. Blood lipids<br>6. changes in medications<br>7. Patient-reported outcomes (PROs)<br>8. PAID, DEF/SWE, DTSQ, HADS, EQ-5D-5L<br>9. eCRF
[58]	Switzerland	Adult patients hospitalised	Home	1. Patient domain	- Self-management	1. all-cause re-admission

		with decompensated heart failure irrespective of left ventricular ejection fraction, and a brain natriuretic peptide and history of dyspnea, increased fatigue or weakness		2. Professional domain 3. Organizational domain	- Patient education - Discharge management - Clinician education - Continuity of care - Disease management	2. Death 3. Quality of life
[59]	Australia	Low income with diabetes and depression	GP clinic	1. Patient domain 2. Professional domain 3. Organizational domain	- Case management - Patient education - Multidisciplinary team - Clinician education - Disease management	1. St George's Respiratory Questionnaire 2. COPD Assessment Test 3. FEV% 4. modified Medical Research Council 5. Hospital Anxiety scale 6.Hospital Depression scale
[60]	Sweden	A partner and patient diagnosed with heart failure recently discharged from hospital	Clinic / home	1. Patient domain	- Discharge management - Self-management - Patient education	1. SF-12 (PCS/MCS)[d] 2. Physical functioning 3. role limitations due to physical health problems 4. Bodily pain

						5. General health<br>6. Vitality<br>7. Social functioning<br>8. Role limitations due to emotional problems<br>9. Mental health<br>10. Beck depressions inventory (BDI)<br>11. Control attitude scale (CAS)
[61]	India, Indonesia, Malaysia, the Philippines, Singapore, Taiwan, Thailand, and Vietnam	Patients with type 2 diabetes	Hospital/ community health centre	1. Patient domain<br>2. Professional domain<br>3. Organizational domain	- Self-management<br>- Patient education<br>- Patient reminders<br>- Clinician education<br>- Clinician reminders<br>- Disease management<br>- Electronic patient registry	1. Diabetes-associated end points<br>2. HbA1c<br>3. Cholesterol<br>4. BP<br>5. BMI[e]<br>6. eGFR

[62]	Taiwan	Heart failure	Hospital/ home	1. Patient domain<br>2. Professional domain<br>3. Organizational domain	- Case management<br>- Integrated care pathway<br>- Discharge management<br>- Multidisciplinary team<br>- Clinician education<br>- Clinician reminders<br>- Disease management<br>- Clinical information transmission	1. Heart failure-related re-hospitalization<br>2. Heart failure disease knowledge (DHFKS)<br>3. 6-min walking distance<br>4. BP<br>5. BMI[e]<br>6. LVEF<br>7. heart rate<br>8. eGFR<br>9. Hb
[63]	China	Patients with diagnosis of type 2 diabetes	Hospital / home	1. Patient domain<br>2. Professional domain<br>3. Organizational domain	- Self-management<br>- Patient education<br>- Patient reminders<br>- Multidisciplinary team<br>- Continuity of care	1. HbA1c<br>2. the Chronic Disease Self-Efficacy Scale<br>3. 36-item Short-Form Health Survey<br>4. Treatment Adherence Scale for Patients with Diabetes

					- Audit and feedback<br>- Managed care programs<br>- Clinical information transmission	
[64]	Israel	Type 2 diabetic patients	Clinic	1. Patient domain<br>2. Professional domain<br>3. Organizational domain	- Case management<br>- Integrated care pathway<br>-Self-management<br>- Multidisciplinary team<br>- Continuity of care<br>- Clinician reminders<br>- Managed care programs<br>- Electronic patient registry	1. Response to treatment<br>2. Compliance<br>3. HbA1c<br>4. Blood glucose<br>5. BMI[e]
[65]	Sweden	Heart failure	Clinic/<br>home/<br>hospital	1. Patient domain<br>2. Professional domain<br>3. Organizational domain	- Case management<br>- Integrated care pathway<br>- Patient education	1. Drug use<br>2. BP<br>3. Serum potassium

					- Multidisciplinary team	4. Serum creatinine
					- Continuity of care	
					- Disease management	
					- Clinical information transmission	
[66]	USA	Type 2 diabetic patients	Clinic	1. Patient domain<br>2. Professional domain<br>3. Organizational domain	-Case management<br>- Integrated care pathway<br>- Patient education<br>- Multidisciplinary team<br>- Continuity of care<br>- Audit and feedback<br>- Disease management<br>- Electronic patient registry (data exchange and interoperability)	1. HbA1c change<br>2. Self-efficacy (DES)<br>3. REALM<br>4. PHQ-9[a]
[67]	Australia	Patients with depression and type 2	Health care	1. Patient domain<br>2. Professional domain	- Case management<br>- Integrated care pathway	1. PHQ-9[a]<br>2. SF-36[d] mental

	diabetes,	practices	3. Organizational domain	- Patient education	3. SF-36[d] physical
	coronary heart disease			- Clinician education	4. BMI[e]
	or both			- Continuity of care	5. Waist (cm)
				- Disease management	6. Systolic blood pressure
					7. Total cholesterol (mmol/l)
					8. LDL-cholesterol (mmol/l)
					9. HDL-cholesterol (mmol\l)
					10. Triglycerides (mmol\l)
					11. HbA1c (%)
					12. 10-year CVD risk
					13. Smoking
					14. Alcohol
					15. Exercises 30 min/day 16. Referred to exercise program 17. Attends exercise programme 18. On Anti-depressant medication 19. Referred to mental health worker 20. Attends mental health worker

[68]	USA	Patients with depression and type 2 diabetes	Hospital/ clinic	1. Patient domain<br>2. Professional domain<br>3. Organizational domain	- Case management<br>- Integrated care pathway<br>- Patient education<br>- Multidisciplinary team<br>- Clinician education<br>- Clinician reminders<br>- Disease management<br>- Electronic patient registry (data exchange and interoperability)	1. HbA1c<br>2. PHQ-9[a]
[69]	USA	Heart failure	Hospital	1. Patient domain<br>2. Professional domain<br>3. Organizational domain	- Case management<br>- Discharge management<br>- Self-management<br>- Continuity of care<br>- Clinician Education<br>-Disease management	1. Nursing skill mix<br>2. Nursing care hours per<br>3. Patient day (HPPD)<br>4. Nurse-turnover<br>5. Practice Environment Scale (PES)

					- Clinical information transmission	
[70]	United Kingdom	Adults admitted to hospital with an exacerbation of COPD in the previous year and who were thus at risk of future admissions	Hospital/ home	1. Patient domain<br>2. Professional domain<br>3. Organizational domain	- Case management<br>- Integrated care pathway<br>- Self-management<br>- Multidisciplinary team<br>- Continuity of care<br>- Audit and feedback<br>- Disease management<br>- Electronic patient registry (data exchange and interoperability)	1. Hospital admission with COPD exacerbation<br>2. All-cause hospital admissions<br>3. SGRQ<br>4. HADS-B<br>5. SECD6<br>6. LINQ<br>7. MARS<br>8. FEV1
[71]	Netherland	Patients diagnosed with type 2 diabetes and treated in primary care	Primary care facility	1. Patient domain<br>2. Professional domain	- Self-management<br>- Clinician education	1. The medication possession ratio<br>2. The 5-item Medication Adherence Rating Scale<br>3. The dispensing date of the next prescription (“drug holidays”), also based on pharmacy refill data and self-reported adherence

[72]	USA	Type 2 diabetes patients	Clinic	1. Patient domain<br>2. Professional domain<br>3. Organizational domain	- Case management<br>- Integrated care pathway<br>- Patient reminders<br>- Continuity of care<br>- Clinician education<br>- Clinician reminders<br>- Disease management<br>- Managed care programs	1. HbA1c<br>2. Cholesterol<br>3. BP<br>4. All-cause hospital visits<br>inpatient days
[73]	USA	Patients with systolic heart failure and comorbid Depression	Hospital /<br>home	1. Patient domain<br>2. Professional domain<br>3. Organizational domain	- Case management<br>- Self-management<br>- Clinician education<br>- Disease management	1. Quality of life (mHRQOL)<br>2. MCS-12<br>3. Patient-Reported Outcomes Measurement Information System–Depression effect size<br>4. Physical function<br>5. HF Pharmacotherapy use<br>6. Re-hospitalisations

						7. Mortality
[74]	Canada	Patients diagnosed with COPD according to GOLD guidelines	Hospital / home	1. Patient domain<br>2. Professional domain<br>3. Organizational domain	- Case management<br>- Patient education<br>- Multidisciplinary team<br>- Clinician education<br>- Continuity of care<br>- Disease management	1. Emergency department visits<br>2. Hospital admissions<br>3. Mortality
[75]	Australia	Participants were referred to the diabetes clinic or hospital	Hospital / clinic	1. Patient domain<br>2. Professional domain<br>3. Organizational domain	- Integrated care pathway<br>- Case management<br>- Multidisciplinary team<br>- Clinician education<br>- Disease management<br>- Electronic patient registry	1. Total cholesterol (mmol/l)<br>2. LDL-cholesterol (mmol/l)<br>3. HDL-cholesterol (mmol/l)<br>4. Triacylglycerol (mmol/l)<br>5. Serum creatinine (mmol/l)<br>6. eGFR<br>7. BMI

						8. SBP (mmHg)<br>9. DBP (mmHg)<br>10. The diabetes-related QoL (DQoL-Brief) survey<br>11. Short-form 12v2-physical<br>12. Short-form 12v2-mental<br>13. Client satisfaction questionnaire score<br>14. Self-management support score<br>15. Doctor visits per participants
[76]	Sweden	Chronic and severe heart failure	Clinic/<br>home/<br>hospital	1. Patient domain<br>2. Professional domain<br>3. Organizational domain	- Case management<br>- Integrated care pathway<br>- Discharge management<br>- Multidisciplinary team<br>- Continuity of care<br>- Disease management	1. Health-related quality of life (QALY)<br>2. Costs of health care

					- Managed care programs	
[77]	Germany	Elderly 60+ with chronic heart failure	Pharmacy	1. Patient domain<br>2. Professional domain	- Patient education<br>- Patient reminders<br>- Integrated care pathway<br>- Continuity of care	1. Medication adherence Beta-blocker<br>2. Medication adherence ACEi/ARB<br>3. Medication adherence MRA
[78]	Singapore	High-risk patients with uncontrolled type 2 diabetes, polypharmacy, and multiple comorbidities	Primary care facility	1. Patient domain<br>2. Professional domain	- Integrated care pathway<br>- Multidisciplinary team<br>- Continuity of care	1. Costs-effectiveness 2. ICER
[79]	USA	Adults with diagnose type 2 diabetes or prediabetes	Community	1. Patient domain<br>2. Professional domain	- Patient education<br>- Clinician education	1. HbA1c<br>2. Total cholesterol<br>3. HDL-cholesterol<br>4. LDL-cholesterol<br>5. Triglycerides<br>6. Systolic BP

						7. Diastolic BP 8. BMI[e]
[80]	USA	Adults with type 2 diabetus	Clinic	1. Patient domain	- Self-management<br>- Patient education	1. HbA1c<br>2. Systolic BP<br>3. Diastolic BP<br>4. Body mass index<br>5. Weight<br>6. B12 therapy<br>7. Statin therapy<br>8. Comprehensive foot exam<br>9. Influenza vaccination<br>10. Pneumococcal vaccination<br>11. Retinal eye exam<br>12. Urine microalbumin
[81]	Netherlands	Stroke patients	Geriatric rehabilitation	1. Patient domain	- Case management	1. Daily activity (FAI)

			stroke units			
				2. Professional domain	- Integrated care pathway	2. Functional dependence (Katz-15)
				3. Organizational domain	- Discharge management	3. Perceived quality of life (SSQoL)
					- Multidisciplinary team	4. Social participation (IPA)
					- Continuity of care	MMSE
					- Clinician education	
					- Disease management	
					- Managed care programs	
[82]	Finland	Heart failure	Hospital/	1. Patient domain	-Case management	1. Number of HF-related hospital days
			home	2. Professional domain	- Integrated care pathway	2. Death from any cause
				3. Organizational domain	- Patient education	3. Heart transplant operation or listing for transplant operation,
					- Multidisciplinary team	4. Left ventricular ejection fraction (LVEF) measured by echocardiography,
					- Continuity of care	5. EHFSBS score
					- Clinician reminders	
					- Disease management	6.plasma concentration of N-terminal of the prohormone brain natriuretic peptide (NT-proBNP, ng/l),

					- Electronic patient registry	7. creatinine (μmol/l),<br>8. sodium (mmol/l)<br>9. potassium (mmol/l)
[83]	USA	Patients with stroke and moderate upper extremity motor impairment	Clinic /<br>home	1. Patient domain<br>2. Professional domain<br>3. Organizational domain	- Patient education<br>- Disease management	1. Long WolfMotor Function Test<br>2. WolfMotor Function Test<br>3. Stroke Impact Scale
[84]	China	Adult stroke patients<br>who chose<br>home recuperation<br>after discharge	Hospital /<br>home	1. Patient domain<br>2. Professional domain<br>3. Organizational domain	- Discharge management<br>- Patient education<br>- Multidisciplinary team<br>- Clinician education<br>- Disease management	1. Fugl-Meyer assessment<br>2. Fugl-Meyer upper extremity<br>3. Fugl-Meyer lower extremity<br>4. Berg Balance Scale<br>5. Timed “Up&Go”<br>6. Six-Minute Walk Test<br>7. Modified Barthel Index<br>8. The Stroke-Specific Quality of Life Scale

[85]	China	Adults with a history of stroke and in stable clinical condition	Clinic / home	1. Patient domain<br>2. Professional domain<br>3. Organizational domain	- Integrated care pathway<br>- Self-management<br>- Multidisciplinary team<br>- Clinician education<br>- Continuity of care<br>- Managed care program	1. Systolic BP measure<br>2. Diastolic BP measure<br>3. EQ-5D-5L<br>4. The short-form International Physical Activity Questionnaire<br>5. Medication adherence in Antiplatelets<br>6. Medication adherence Statins<br>7. Medication adherence Antihypertensives<br>8. Stroke recurrence<br>9. Stroke hospitalization in the past year<br>10. Moderate to severe disability<br>11. Death<br>12. Time Up and Go
[86]	China	Patients diagnosed with COPD or	Clinic	1. Patient domain<br>2. Professional domain	- Case management<br>- Discharge management	1. Forced expiratory volume in 1 s<br>2. Forced vital capacity

		were found to have a high risk of COPD by physical examination and spirometry testing		3. Organizational domain	- Patient education<br>- Continuity of care<br>- Clinical education<br>- Multidisciplinary team<br>- Disease management	
[87]	China	Patients with ischemic stroke within seven days of onset	Hospital / clinic	1. Patient domain	- Discharge management	1. The Fugl-Meyer Assessment-upper limbs<br>2. The Fugl-Meyer Assessment-lower limbs<br>3. National Institutes of Health Stroke Scale<br>4. Barthel index

[a]PHQ-9: Patient Health Questionnaire-9 score

[b]SCL-20/90/D13: Symptoms Checklist Depression Scale 20 items or 90 items or core depression subscale

[c]MMSE: Mini-mental state examination

[d]SF-36/12: Short Form Health Survey 36 items or 12 items

[e]BMI: Body Mass Index

[f]EQ-5D/-5L: EuroQol five-dimension scale questionnaire

[g]PSS-4: Perceived Stress Scale 4

[h]HADS: Hospital Anxiety and Depression Scale

[i]HAF-17: Herzangstfragebogen

[j]HRQoL: Health-related quality of life

[k]GSE-6: The General Self-Efficacy Scale

[l]ESSI: The ENRICHD Social Support Instrument

[m]ESAS: The Edmonton Symptom Assessment Scale

[n]KCCQ: Kansas City Cardiomyopathy Questionnaire

[o]WHO-QOL/-BREF: World Health Organization Qualitiy of Life Questionnaire

[p]GAD-7: General Anxiety Disorder-7

[Q]PACIC: The Patient Assessment of Chronic Illness Care

[r]CES-D: Centre for Epidemiological Studies Depression Scale

Table 4. Characteristics of the integrated care interventions included in the systematic review and meta-analysis.

Study	Intervention	Control
[23]	In addition to the usual care, patients in the intervention group received a clinical pharmacist service. Pharmacists and physicians worked collaboratively at the same time and setting. Clinical data, data on pharmacotherapy, information on lifestyle, and sociodemographic data were collected from the patients or other sources available to the pharmacist. The analysis of such information guided the process of building the necessary pharmacist interventions. During face-to-face consultations, health education-related issues were discussed, and specific information was provided about the use of medications. The clinical pharmacist also conducted a comprehensive medication review to identify drug-therapy problems.	Participants in the control group received only the usual care services.
[24]	Patients randomized to the intervention group of self-management support from non-physician care coordinators, decision support electronic health records facilitating physician treatment adjustments, and specialist case reviews; teams and care coordinators at each site received a 3-day in-person training.	Usual care
[25]	The intervention group in each practice was exposed to the virtual clinic following a standardized protocol, clinical review by primary care and specialist diabetes teams; assessment of clinical needs (risk factors, clinical data, complications and other comorbidities) and therapy review (level of optimization); formulation of a clinical management plan (therapy changes or adjustments; lifestyle areas and targets; and individualized clinical targets); and a follow-up patient consultation to agree an individualized care plan in partnership with the patient.	The control condition was usual diabetes care according to local and national diabetes pathways.
[26]	The specialized multidisciplinary diabetes program included a visit to the certified diabetes educator/nurse practitioner who developed an individualized plan for every subject. Subsequent visits were with a nurse practitioner, nutritionist,	Patients in the control group returned to their pre-hospitalization outpatient care.

	social worker and endocrinologist as needed.	They were not discouraged to consult a specialist or diabetes educator as part of their care.
[27]	Integrated care intervention in which the integrated care manager collaborated with physicians to offer education and guideline-based treatment recommendations to patients and to monitor adherence and clinical status.	Usual care
[28]	The intervention RECODE focused on implementing more efficient COPD care. The 20 teams of the intervention group were trained in essential components of effective COPD-DM: proper diagnosis, optimizing medication adherence, motivational interviewing, smoking cessation counselling, applying self-management plans including early recognition and treatment of exacerbations, physical (re)activation, and nutritional support. In addition, the teams learned the details of a web-based computer program for measuring and reporting process and outcome performance indicators, named ZORGDRAAD.	The control group of providers team provided usual care
[29]	Each care manager contacted his/her participants regularly to: (a) review their risk factors and set individual goals for lifestyle changes with each patient using shared-decision making; (b) support them in adhering to treatment plans, which were discussed during the first intervention contact and conformed to national guidelines; (c) monitor progression and coordinate care; and (d) connect them with self-help groups (e.g., for smoking cessation) and other support resources (e.g. psychotherapy)	Waiting
[30]	Patients in the intervention group were offered a multidisciplinary approach involving collaboration between specialists in palliative and heart failure care, i.e. specialized nurses, palliative care nurses, cardiologist, palliative care physicians, physiotherapists, and occupational therapists. The patients were also offered structured, person-centered care (PCC) at home. The intervention was carried out as follows: (i) after identifying a patient who fulfilled the inclusion criteria and had no exclusion criteria earlier described in the design paper, a responsible physician and nurse were identified for each patient; (ii) the patient was then called for a thorough medical examination by the responsible a physician with identification of co-morbidities and assessment of physiological, social, and spiritual needs; followed by (iii) meeting	The control group received care as usual.

[31]	Participants attending practices in the collaborative care arm were offered a choice of appropriate evidence-based low-intensity psychological treatments, delivered over 3 months through IAPT services.	Participants attending practices allocated to usual care received standard management from their primary care team
[32]	The intervention group received TECHNOB program consisting of two phases 1) inpatient phase where participants attend an intensive four week and medically-managed program for weight reduction and rehabilitation; 2) outpatient phase where patients receive armband to monitor calories burned, active energy, physical activity duration and levels. This information is shared with the physicians for monitoring. Additionally, they had access to TECHNOB web-platform which has several functions and delivers many utilities, such as questionnaires, an animated food record diary, an agenda and a videoconference virtual room for contact with the dietitian. Dietitian sends also SMS in order to visually display the food choices (frequency and portions) outpatients have to adhere according to dietary prescriptions.	Patients assigned to control group did not receive any instructions and were discharged as all the other inpatients not included in the study
[33]	The management group received the integrated health management model including the following components: (1) Health record establishment, which was implemented by specifically-trained community health service centre staff; (2) Health evaluation, which was done by related researchers through the health evaluation software; and (3) Health management, including diet advice, individual psychological aspects of health, a tailor-made exercise program based on an earlier evaluation, education/skills training on health self-management, individual telephone consultation, group lectures on health and diabetes, follow-up visits, regular blood glucose monitoring, long-term diabetes drug monitoring and distribution of health-promoting materials, which were implemented by specifically-trained community health service centre staff, managers and related researchers.	The control group received care as usual.
[34]	Patients in the MDMP intervention group were followed by the HF team consisting of 3 cardiologists, 1 coach nurse, 10 nurses, 1 dietitian, and 1 psychiatrist, and received a multidisciplinary intensive intervention in addition to standard care. This intervention included enhanced discharge education, physical exercise training, and follow-up contacts.	The control group received care as usual.

[35]	The home THC platform is a comprehensive solution for the care and monitoring of chronic patients, modelled and tested in patients with CHF that enables the provision of multichannel service and patient tracking through patient monitoring of biometric data (weight, heart rate and blood pressure), symptoms reporting (seven questions to capture worsening symptoms of the cardiac condition, mainly worsening heart failure, and one question to capture general worsening), generation and management of warning alarms (biometrics out of range) and alerts (information related to the function of the household devices).	Face to face encounter
[36]	Participants in the collaborative care arm received up to eight face-to-face sessions of brief psychological therapy delivered by a case manager who were "psychological wellbeing practitioners" employed by Improving Access to Psychological Therapies services in the English NHS.	Usual care
[37]	The intervention was envisioned as an integrated care delivery model in primary care, bringing together active behavioural intervention with diabetes medical care in the same setting. The intervention was designed to be tailored or severity stratified such that intervention arm patients were assigned to one of two levels of behavioural treatment based on the level of their baseline distress and depression, small changes lifestyle coaching or CBT. The CBT subgroup intervention focused on the reduction of depressive and/or RRD symptoms through modification of negative thoughts and problematic behaviours as well as improvement of diabetes self-management strategies. CBT intervention components were guided by two evidence-based treatment manuals for behavioural activation.	The control group received standard medical care.
[38]	For the intervention group, a multidisciplinary post-stroke consultation team was developed to provide integrated transitional care (TC). A home visit is the key component for the intervention group over the next eight weeks. As soon as the patient was discharged to home, the team was contacted by the designated staff at the stroke unit.	The control group received usual post-discharge care by the staff from community healthcare stations. Detection and control of risk factors and medication therapy based on secondary

	The team’s activities during the home visits include (1) ongoing stroke rehabilitation performed by rehabilitation therapists, (2) medication reconciliation performed by general practitioners and nurses and (3) self-management education regarding risk.	stroke prevention strategy.
[39]	Patients randomized to the intervention group were scheduled for an outpatient clinical review with the study team within 2 weeks of hospital discharge. At this initial clinic visit the patient’s clinical status was reviewed, with particular attention to possible remediable exacerbating factors. A follow-up plan was devised for each patient aiming for 6-weekly visits alternating between the GP and heart failure clinic, although the patients were free to see their GPs at any time they wished.	Patients randomized to the control group continued under the care of their GP with additional follow-up measures as usually recommended by the medical team responsible for their in-patient care.
[40]	Patients in the intervention group were referred to a multidisciplinary specialized heart failure outpatient clinic at the Montreal Heart Institute, where they were evaluated by the study team within 2 weeks of hospital discharge. At the clinic, the patient could be evaluated both clinically and para-clinically, receive intravenous diuretics if required and be observed for up to 5 hours. Additional to these services, a nurse telephoned all patients in the intervention group within 72 hours of hospital discharge and then monthly, unless a problem occurred that required more frequent contact. After the baseline evaluation, the clinic cardiologists individualized a treatment plan for each patient in the intervention group.	Received treatment and appropriate follow-up according to the standards of the attending physicians (all staff cardiologists of the same university hospital) but without further direct contact with the research team or the

		planned intervention.
[41]	The intervention group received Integrated HF-diabetes Self-Care intervention. participants in the intervention group participated in individualized education and counseling session. Family members were encouraged to attend. A trained research nurse provided an overview of the content using a semi-structured script and coordinated set of PowerPoint illustrations viewed on a laptop computer.	The control group received care as usual.
[42]	1. maximize recruitment and minimize attrition;<br>2. enhance patient depression treatment engagement and adherence;<br>3. reduce individual, provider, and system barriers to depression and diabetes care via the provision of system and community resource navigation;<br>4. integrate depression and diabetes care; and<br>5. provide culturally and linguistically competent depression care.	Standard clinic care
[43]	The HCISM intervention included several steps: 1) The integrated intervention group was established. 2) hospital-community information platform to share and discuss patient information. 3) Hospital nursing group for the formulation of specific intervention plan based on patient's condition in the hospital. 4) Nursing group of the community steps in after	The control group received the usual discharge routine intervention consisting of rehabilitation knowledge, training guidance, medication and telephone

	the discharge of the patient to perform the intervention rehabilitation plan	follow-up once a month
[44]	The intervention group received KM2H2 program. KM2H2 consists of six intervention sessions and two booster sessions. The six intervention sessions include two lectures, two sessions of telephone counselling, and two group meetings, all delivered on a weekly basis. The intervention was delivered by 35 trained interventionists, five were doctors in preventive medicine from Wuhan CDC and the rest were CHC physicians. The interventionists all received one-week fulltime training.	The control group received the standard CBHCP care.
[45]	The Educoeur program of lifestyle modification was conducted by nutritionists, psychologists and kinesiologists with follow-up by nurses and physicians. Patients received 12 weekly group sessions of 3 h between months 3 and 6 of the study. Every 3-h group meeting was divided in 3 educational and experimental sessions focusing equally on nutrition, physical activity and stress management/motivation. Patients were therefore exposed to 12 h in nutrition, in physical activity and in stress management.	1. Specialized care intervention<br><br>Participants were referred to a physician specialized in cardiovascular prevention at the IRCM. The frequency of their medical follow-ups was not fixed by the study protocol but instead determined by their health status. When judged necessary, the specialist could refer to the staff nutritionist for assessment and follow-up.<br><br>2. Usual care intervention<br><br>Participants were referred to their family physician, providing the results of their blood work along with medical recommendations and informing them they would be called at 2 years for a final assessment
[46]	Nurse-led outpatient care steered by decision support software based on the guidelines and supervised by a cardiologist.	Usual care by a cardiologist in the outpatient clinic during visits scheduled to last 20 min for the first visit and 10

		min for follow-up visits
[47]	The intervention group received a program consisting of: 1) A comprehensive assessment of the patient at entry, 2) A 2-h educational programme was administered at entry by a respiratory nurse, 3) One joint visit of the specialised nurse and the primary care team (physician, nurse and social worker) at the patient home was completed within 72 h after entry into the study. (4) Accessibility to the specialised nurse at the hospital was ensured for primary care professionals during the follow-up period.	The control group received usual care
[48]	The 3-month multidisciplinary supportive program included a printed brochure developed for caregivers, three 60-min sessions of group classes, three 30-min peer support group sessions, and regular telephone-based consultations and follow-up during the program	Control group received usual care
[49]	Patients in the intervention group met with the pharmacist at their respective primary care site for an assessment of adherence, barriers to optimizing blood glucose levels, and current medication regimen. All intervention patients received individualized education regarding diabetes self-management, including diet, exercise, blood glucose level testing, medications, and insulin. The pharmacist followed guidelines of the Management of Hyperglycemia in Type 2 Diabetes.	Control group included the use of registries and targeted patient outreach
[50]	The intervention group received a Nurse-led community-based multidisciplinary program (CNMP) that consisted of individual assessment, group health education, individual consultation, and follow-up. For a nurse-led multidisciplinary team construction, we included team members from local health facilities and researchers: registered nurses, physicians, dieticians, physiotherapists, and volunteer patients. The plan could include encounters with one or more professionals in the disciplines of nursing, medicine, physiology, and nutrition. After completing a comprehensive individualized assessment, the nurses shared it with the multidisciplinary team. The intervention plan could be further adapted by any participating professional in those disciplines.	The participants in the control groups received the routine care provided by the community health care centre
[51]	Telemedicine consisted of the same number of pre-specified, structured follow-up encounters, but these were performed remotely rather than on site, using videoconference or audioconference. In these remote visits, the interventions were	Usual care' consisted of several pre-specified, on-site, face-to-face,

	identical to those conducted in the usual care arm, including health education and pharmacological up-titration interventions ('teleintervention'). Patients were instructed to self-monitor their bio-measures on a daily basis and register this information together with any HF signs and symptoms in an user-friendly software, which transferred this data immediately to the HF unit ('tele-monitoring').	structured follow-up encounters, and patients were instructed to self-monitor their bio-measures on a daily basis and contact<br><br>the nurses in the event of any abnormality (e.g. weight<br><br>gain, incipient decompensation signs)
[52]	The selected community stroke rehabilitation team undertook training on theory, research, and practical application of the Bridges self-management program. Patients of the selected rehabilitation team were introduced to the stroke workbook and the seven key principles of self-management.	The control group selected community stroke rehabilitation teams provided patients with rehabilitation as usual
[53]	The intervention team consisted of a cardiologist, a CHF nurse, a telephone nurse coordinator, and the patient's primary physician. The CHF cardiologists designed and documented a treatment plan for all study patients before randomization. Contact with the patient was on a pre-specified schedule via phone call by the CHF nurse.<br><br>The CHF nurse followed an algorithm to adjust medications.	Patients in the non-intervention group were followed as usual by their primary physicians.
[54]	In the intervention group care facility has access to full care tasks such as screen on the first visit, diagnose, and maintain patient records; use provided desk guide on how to prescribe, educate, follow-up, and retrieve patients	The control group care facilities has access only to limited care tasks: screen on the first visit, diagnose, and maintain patient records only
[55]	The intervention group received an integrated diabetes self-management program using a smartphone app based on the IMB model. The present study's intervention was developed to increase knowledge about diabetes self-management via face-to-face education and to improve self-management motivation and behaviour skills via a diabetes self-management smartphone app and phone counselling. To improve the content validity of the diabetes self-management education, this	The control group received a book entitled Diabetes Management Guide published by the Korea Association of Diabetes Nursing Education.

	study engaged in content validity testing by expert groups, each comprising five individuals: two endocrinologists, one diabetes nurse educator, one diabetes education nutritionist, and one diabetes education pharmacist.	
[56]	The intervention group received self-management action plans, including early recognition and treatment of exacerbations, encouragement of regular exercise and guideline-based physical reactivation, cooperation with secondary care, and instructions in nutritional support. The secondary aim of the course was to provide a network platform for team members.	The control group received usual care
[57]	The Intervention group will follow the integrated PDM process along with the usual care. PDM consists of structured SMBG regimen based on each patient's treatment regimen and Accu-Check Smart Pix diabetes management system providing visualization of blood glucose measurement results and therapy data from compatible glucose monitoring systems	Control group patients will continue usual treatment according to their customary medical routine
[58]	The intervention group received the intervention once the patients were discharged to home. Intervention patients received one home visit by a specialized HF nurse approximately 1 week after returning home after discharge from either hospitalization or rehabilitation, followed by 17 telephone calls in decreasing intervals over the next 12 months.<br><br>The home visit consisted of a physical, psychosocial, and environmental assessment, the provision of educational, behavioural, and supportive care to build self-care abilities, and individualized patient goal-setting to increase self-efficacy. All intervention group patients were given a special kit published by the Swiss Heart Foundation that included in-depth explanations of HF and self-care procedures. Following the home visit, an individualized nursing care plan was developed that included the patient-identified goals and the goals that the nurse identified based on the results of the assessments. This plan was then discussed with the primary care physician to elicit his/her support and to coordinate and prioritize goals.	The control group received care as usual.
[59]	The intervention group received RADICALS model of care consisting of training on spirometry and the COPD-X guidelines for the providers. For patients a smoking cessation support, home medicines review, eight-week home-based pulmonary rehabilitation program was available.	The control group received usual care and received a booklet for COPD

[60]	The patient-partner in the intervention group received care as usual. In addition, they participated in an educational and psychosocial intervention, which included psychosocial support to maintain and strengthen the dyads' physical and mental functions and perceived control. The intervention was delivered in three modules through nurse-led face-to-face counselling, a computer-based program and written materials. The sessions took place two, six and twelve weeks after discharge from hospital.	The control group received care as usual.
[61]	The intervention group received 3 care components: a nurse-led Joint Asia Diabetes Evaluation (JADE) technology-guided structured evaluation, automated personalized reports to encourage patient empowerment, and 2 or more telephone or face-to-face contacts by nurses to increase patient engagement.	In phase 1, the control group received the JADE technology–guided structuredevaluation and automated personalized reports. In phase 2, the control group received the JADE technology–guided structured evaluation only.
[62]	The multidisciplinary disease management program (MDP) included comprehensive assessments, individualized education, optimizing medications, pre-scheduled clinic visits, and encouraging regular physical activity at home. MDP with exercise training included outpatient-based exercise training and home exercises. MDP without exercise training performed only home exercises.	In the control group, the primary care cardiologist was responsible for the medical treatment, evaluation, symptom control, and clinical visits. No heart failure nurse specialist was involved.
[63]	The participants in the intervention group received web-based transitional care. The web-based transitional care program was designed following a comprehensive literature review and consultation with experts (nurses who specialize in diabetes, endocrinologists, dietitians, and rehabilitation therapists). The program consists of five modules: disease self-management (diet, exercise, medication management, and blood glucose self-monitoring), health education, group	The participants in control group received the usual care including provision of a routine discharge education and a diabetes knowledge manual diabetes knowledge manual

	interaction, remote counselling, and data collection.	before discharge.
[64]	Patients from the intervention clinic received the interdisciplinary approach offered by WNMCDP. According to the clinic protocol, all patients are examined by the physician, receive dietary counselling and have a session with the diabetes nurse educator. Continuation of treatment and follow-up visits are scheduled according to the patient's condition.	Patients continued usual treatment offered by the doctors and nurses
[65]	The intervention group appointed physician and nurse for each patient and the patient was then called for a thorough medical examination with identification of comorbidities and assessment of physiological, social and spiritual needs followed by a meeting with nurses who used a model for person-centred palliative care. Patients in the control group were managed by their responsible physicians primarily at the primary healthcare centre or at the hospital as appropriate.	Patients in the control group were managed by their responsible physicians.
[66]	Patients randomized to the intervention arm continued to receive usual care, and were also provided access to a nurse case manager and a disease management module. They received an initial 1-h consultation with the case manager and one-on-one instruction with the trial's Web module from the study coordinator. Following the initial clinic visit, all remaining intervention activities took place remotely via e-mail and Web resources.	Participants randomized to the control arm received usual care from their DCC practice team.
[67]	As part of the TrueBlue model, patients were scheduled to visit the practice every 3 months for a 45 min nurse consult followed by a 15 min consult with their usual GP, in which stepped care (psychotherapy or pharmacotherapy) was offered if depression scores had not improved or had not dropped below a value of 5.<br>The PN used the care-plan template and obtained current physical measures and reviewed recent pathology results. PNs also reviewed lifestyle risk factors.	Wait-list control group

[68]	Patients in the intervention group received a 6-month blended diabetes and depression behavioural health coaching program, followed by a 6-month maintenance period without coaching (intervention). Healthy Outcomes Through Patient Empowerment (HOPE) included 9 telephone sessions with 24 trained health care professionals using collaborative goal-setting and behavioural activation methods.	Control group received usual clinical care plus educational materials (EUC) for 12 months.
[69]	The quality collaborative intervention included a 2-day in-person meeting, an evidence-based HF tool kit, and monthly group teleconference calls with the site coordinators and study team. The HF toolkit included resources that could be tailored for implementation in each organization (e.g., fact sheet, education modules, discharge checklist, patient education).	Patients in the control group were managed as usual for 6 months and were then switched to secondary intervention group.
[70]	Participants randomized to receive tele-monitoring recorded a daily questionnaire about symptoms and treatment use, and monitored oxygen saturation using linked instruments. Algorithms, based on the symptom score, generated alerts if readings were omitted or breached thresholds. They also received similar care from existing clinical services.	The control group was provided with the usual clinical care.
[71]	The intervention group received a PRISMA program consisting of two sessions with a dietician and a practice nurse both experienced in diabetes care. These trainers had followed a standardized training program to ensure the quality of information delivery. The philosophy of PRISMA is based on patient empowerment, grounded in the following four psychological models: the self-regulation theory, the dual process theory, the self-determination theory, and the social learning theory.	Wait-list control group
[72]	Intervention patients received 12 months of Web-based care management. The Web-based program included patient access to electronic medical records, secure e-mail with providers, feedback on blood glucose readings, an educational Web site, and an interactive online diary for entering information about exercise, diet, and medication	Usual care

[73]	The intervention included three different groups: A) Blended With depression: our blended care manager team provided collaborative care for depression. Specifically, they (1) inquired about participants' psychiatric history; (2) provided basic psychoeducation; (3) assessed treatment preferences for depression; and (4) monitored mood symptoms with the PHQ-9. B) Without depression blended: Same intervention only for participants without depression. C) Enhanced UC: Based on participants' clinical status, treatment preferences, and their case review discussions with the study cardiologist (R.R.), the care managers typically encouraged (1) adherence to guideline-recommended HF pharmacotherapy; (2) healthy lifestyle (e.g., physical activity, tobacco cessation, and other HF self-care); (3) maintaining weight within a narrow range; and (4) keeping follow-up medical appointments.	The control group received usual care.
[74]	The intervention group received a multi-component, case manager-led intervention including 1) case-manager delivered 40-min standardized education session based on Living Well with COPD on study enrolment; 2) individualized care and action plans for COPD exacerbation recognition, self-management, and management of comorbidities; 3) case manager-initiated telephone consultations comprising standardized reinforcement/motivational interviewing focusing on health behaviours; action plan teach-back sessions; assessment of symptoms/symptom monitoring, problems, and problem-solving strategies; 4) ongoing case manager communication with family physicians and with hospital specialists including respirologists; and 5) priority access to ambulatory outpatient clinics. Exacerbation management prescriptions were provided with the action plan either directly to the participant or to their pharmacy.	The control group received usual care.
[75]	The intervention arm comprised a multidisciplinary team including two GPwSIs, an endocrinologist, and a DNE co-located in a community-based general practice. Participants were screened by this team, the DNE reviewed the diabetic history, assessed feet, screened for depression, and took anthropometric measures, by a GPwSI an individualized diabetes management plan was developed to address blood glucose control, BP and lipid control, diabetes complications, lifestyle factors, and any other participant concerns. The endocrinologist then reviewed and endorsed the management plan and, if necessary, co-consulted with the individuals and the GPwSI. Medical reviews, usually 3 monthly, were conducted by the GPwSI and endocrinologist over the course of treatment.	The control group received usual care by hospital-based diabetes specialist

[76]	The patients were offered structured person-centred care at home with easy access to care, and the team was responsible for the total care, including co-morbidities. A model for person-centered palliative care was used. The model is called the six S and consist of the six S key words: self-image, self-determination, social relationships, symptom control, synthesis, and surrender. The PREFER intervention was developed from the goals and steps in the process of providing palliative care for patients with CHF as recommended by the ESC.	The control group (CG) received standard care that is usually provided by a primary health care center or the nurse-led heart failure clinic at the hospital.
[77]	The intervention consisted of the following components: first, medication review (Type 2a according to the Pharmaceutical Care Network Europe (PCNE) classification11) in the community pharmacy at baseline with the aim of generating a consolidated medication plan. Based on the subsequently consolidated medication plan, the patient received a weekly dosing aid together with a printout of the medication plan. The type of the dosing aid (dosette, pill-box) was at the discretion of the pharmacist and in agreement with the patient.	Patients in the usual care group continued to visit pharmacies of their choice to fill prescriptions without further intervention
[78]	In the intervention arm, physicians referred their patients to diabetes nurse educators or dieticians as needed, while clinical pharmacists followed up every four to six weeks with all patients via face-to-face visit or telephone call. In the collaborative management of patients with diabetes, the activities of the clinical pharmacists, diabetes nurse educators and dieticians included but were not limited to provision of medication optimizations, motivational counselling on self-care practices, and advice on nutrition therapy, respectively.	The control group received usual care.
[79]	The intervention group received weekly 3-hours comprehensive diabetes group visits with community health workers integrated as part of the leadership team. Additionally, health workers called or sent text messages to patients regarding weight loss, diet and medication adherence, and reminders	The control group received care as usual.
[80]	The intervention group received a clinical intervention program consisting of community health workers- participant communication, diabetes-led group visits, and physician diabetes training and support via telehealth. During the group visits group discussed vital signs, large group education, three small groups (n = 30 min/section), and a healthy meal. The three small groups addressed medical, social, and behavioural barriers to care. The large group topics focused on diabetes self-management and standards of care	Wait-list control group

[81]	The integrated programme consisted of three care modules; 1) inpatient neurorehabilitation treatment; 2) home-based self-management training for patient and informal caregiver; and 3) stroke education for patient and informal caregiver. The treatment progress was evaluated in monthly multidisciplinary team meetings for every individual patient. All communication and information by the care professionals about the patient and informal caregiver was conducted by using a shared electronic patient record.	The follow-up care is usually provided by monodisciplinary community services, with no multidisciplinary approach.
[82]	Patients regularly reported their most important health parameters to the nurse using a mobile phone app. At the beginning of the study, the patients were given a home-care package including a weight scale, a blood pressure meter, a mobile phone, and self-care instructions. The patients were advised to carry out and report the measurements together with the assessment of symptoms once a week.	Control patients received multidisciplinary treatment according to standard practices.
[83]	The intervention group received Accelerated Skill Acquisition Program (ASAP). This program included initial evaluation and 30 one-hour treatment sessions. Support for patients' control or autonomy was provided by choices of specific tasks to be practiced, collaborative problem solving to identify and address movement needs, and encouragement of self-direction in extending the practice to community contexts.	The control group consisted of Usual and customary care (UCG) and dose-equivalent usual and customary case (DEUCG) based on the usual and customary practice as determined by the therapist.
[84]	After discharge, patients in the intervention group received home remote rehabilitation based on a collaborative care model. Rehabilitation therapists assess the extent of patient dysfunction and work with family caregivers to develop rehabilitation plans and goals.	The control group received routine early rehabilitation guidance and routine nursing measures. After discharge patients received routine care.
[85]	The intervention included both provider-side components and patient-facing components and were supported by a digital health system consisted of an Android-based smart phone application—SINEMA App—for providers and linked with a voice messages system for patients	The control group received usual care.

[86]	The intervention consisted of an intensive intervention phase and an active maintenance phase. In the intensive intervention phase, a respiratory specialist from the authors' hospital performed specific interventions for 1 month or more, at 6-month intervals. In the active maintenance phase, the interventions were mainly performed by local health personnel who received regular supervision from a respiratory specialist and several public health experts from the authors' hospital. In the maintenance phase the participants received information regarding: 1) Systematic health education, 2) Smoking cessation, 3) Management of COPD, 4) Pulmonary rehabilitation at 6-month intervals. In the active maintenance phase, these were mainly performed by local health personnel who received regular supervision from a respiratory specialist and several public health experts from the authors' hospital. In the maintenance phase participants received information regarding: 1) Systematic health education, 2) Smoking cessation, 3) Management of COPD, 4) Pulmonary rehabilitation.	The control group received the usual conventional rehabilitation program.
[87]	The intervention group received Integrated Rehabilitation Techniques of Traditional Chinese Medicine (IRT-TCM) consisting of acupuncture and massage treatmentmainly performed by local health personnel who received regular supervision from a respiratory specialist and several public health experts from the authors' hospital. In the maintenance phase participants received information regarding: 1) Systematic health education, 2) Smoking cessation, 3) Management of COPD, 4) Pulmonary rehabilitation	The control group received usual conventional rehabilitation program .
[87]	The intervention group received Integrated Rehabilitation Techniques of Traditional Chinese Medicine (IRT-TCM) consisting of acupuncture and massage treatment	The control group received usual conventional rehabilitation program

Table 5 The effect of clusters of integrated care.

	CLUSTER 1: Patient empowerment	CLUSTER 2: Network care coordination	Cluster differences (*p* value)
**Total studies**	22 (5093)	32 (7883)	
**OUTCOME MEASURES**			
**Primary outcomes**			
All-cause mortality	1 (1226)<br>RR 1.72<br>[0.82;3.58]<br>I^2: -	5 (1455)<br>RR 1.66<br>[1.10;2.49]<br>I^2: 0%	.93

All-cause hospital admissions	1 (1226)<br>RR 2.10<br>[1.35;3.27]<br>I^2: -	5 (1455)<br>RR 1.56<br>[1.38;1.76]<br>I^2: 7%	.20
Adverse events	1 (1226)<br>RR 2.10<br>[1.02;3.27]<br>I^2: -	3 (1133)<br>RR 1.68<br>[0.61;4.68]<br>I^2: 72%	.64
Healthcare use	-	3 (297)<br>SMD 0.30<br>[-0.50;1.10]<br>I^2: -	-

**Secondary outcomes**			
Quality of life	6 (1665)<br>SMD 0.76<br>[-0.16;1.68]<br>I^2: 90%	9 (2793)<br>SMD 0.13<br>[0.03;0.24]<br>I^2: 0%	.08
Physical functioning	10 (1461)<br>SMD 0.20<br>[0.05;0.36]<br>I^2: 24%	9 (2444)<br>SMD 0.13<br>[-0.14;0.40]<br>I^2: 78%	.60
Weight management	4 (331)<br>SMD 0.05<br>[-0.09;0.19]	6 (1264)<br>SMD 0.10<br>[-0.18;0.37]	.69

	I^2: 0%	I^2: 72%	
Mental health	6 (648)<br>SMD 0.255<br>[-0.16;0.66]<br>I^2: 76%	14 (3754)<br>SMD 0.31<br>[0.10;0.51]<br>I^2: 82%	.76
Self-management	5 (446)<br>SMD 0.49<br>[0.09;0.89]<br>I^2: 44%	8 (2345)<br>SMD 0.20<br>[-0.05;0.45]<br>I^2: 74%	.11
**Tertiary outcomes**			
HbA1c	7 (613)<br>SMD 0.43	8 (1199)<br>SMD 0.48	.85

	[0.10;0.75]<br>I^2: 55%	[-0.10;1.06]<br>I^2: 92%	
Cholesterol	3 (247)<br>SMD 0.03<br>[-0.21;0.27]<br>I^2: 0%	3 (719)<br>SMD -0.04<br>[-0.29;0.20]<br>I^2: 0%	.39
Pulmonary measures	-	4 (1498)<br>SMD 0.17<br>[-0.16;0.49]<br>I^2: 48%	-
Blood pressure control	6 (2012)<br>SMD 0.17	5 (984)<br>SMD 0.04	.21

	[0.01;0.32]	[-0.19;0.27]
	I^2: 7%	I^2: 34%

Blank cells indicate no data available for variables.

Results are represented as number of studies, number of participants, standardized mean differences (SMD) or risk ratios (RR), 95% Confidence interval and Restricted Maximum Likelihood method (I^2) statistics.

Table 6 The effect of integrated care for DMT2, CVD and CRD.

	DMT2	CVD	CRD	Multiple comorbidities	Group differences (P value)
**Total studies**	13 (1457)	22 (4803)	7 (3855)	12 (2851)	
**OUTCOME MEASURES**					
**Primary outcomes**					
All-cause mortality	-	6 (2681)<br>RR 0.60<br>[0.44;0.81]<br>I^2: 0%	-	-	-
All-cause hospital	-	6 (2681)	-	-	-

admissions		RR 0.63<br>[0.56;0.71]<br>I^2: 7%			
Adverse events	1 (305)<br>RR 1.52<br>[0.34;6.76]<br>I^2: -	3 (2054)<br>RR 0.47<br>[0.23;0.96]<br>I^2: 25%	-	-	.09
Healthcare use	2 (174)<br>SMD 0.10<br>[-0.76;0.98]<br>I^2: 0%	1 (93)<br>SMD 0.66<br>[-0.50;1.10]<br>I^2: -	-	-	.01*
**Secondary outcomes**					

Quality of life	3 (511)<br>SMD 0.44<br>[-0.41;1.29]<br>I^2: 75%	6 (1884)<br>SMD 0.65<br>[-0.36;1.67]<br>I^2: 93%	4 (1610)<br>SMD 0.11<br>[-0.07;0.30]<br>I^2: 0%	2 (453)<br>SMD 0.12<br>[-0.08;0.32]<br>I^2: 0%	.21
Physical functioning	3 (479)<br>SMD 0.09<br>[-0.26;0.43]<br>I^2: 0%	11 (1494)<br>SMD 0.30<br>[0.10;0.51]<br>I^2: 63%	2 (1241)<br>SMD -0.13<br>[-1.07;0.80]<br>I^2: 0%	3 (691)<br>SMD 0.04<br>[-0.15;0.23]<br>I^2: 0%	<.01*
Weight management	5 (612)<br>SMD 0.04<br>[-0.16;0.24]<br>I^2: 0%	1 (124)<br>SMD 0.11<br>[-0.24;0.46]<br>I^2: -	1 (288)<br>SMD 0.49<br>[0.26;0.72]<br>I^2: -	3 (571)<br>SMD -0.07<br>[-0.34;0.20]<br>I^2: 0%	<.01*

Mental health	3 (630)<br>SMD 0.21<br>[-0.06;0.47]<br>I^2: 0%	8 (922)<br>SMD 0.42<br>[0.01;0.83]<br>I^2: 85%	3 (1446)<br>SMD 0.14<br>[-0.26;0.53]<br>I^2: 13%	6 (1404)<br>SMD 0.26<br>[-0.07;0.60]<br>I^2: 86%	.50
Self-management	4 (556)<br>SMD 0.36<br>[0.22;0.50]<br>I^2: 0%	5 (485)<br>SMD 0.52<br>[0.03;1.01]<br>I^2: 69%	2 (1291)<br>SMD 0.04<br>[-0.05;0.13]<br>I^2: 0%	2 (459)<br>SMD 0.02<br>[-3.04;3.09]<br>I^2: 82%	<.01*
**Tertiary outcomes**					
HbA1c	9 (943)<br>SMD 0.36<br>[0.10;0.62]	1 (124)<br>SMD 0.08<br>[-0.27;0.43]	-	5 (745)<br>SMD 0.69<br>[-0.32;1.69]	.59

	I^2: 59%	I^2: -		I^2: 94%	
Cholesterol	3 (428)<br>SMD 0.01<br>[-0.17;0.20]<br>I^2: 0%	1 (124)<br>SMD 0.07<br>[-0.28;0.42]<br>I^2: -	-	2 (414)<br>SMD -0.08<br>[-1.00;0.83]<br>I^2: -	.45
Pulmonary measures	-	-	4 (1498)<br>SMD 0.17<br>[-0.16;0.49]<br>I^2: 48%	-	-
Blood pressure control	5 (617)<br>SMD 0.13<br>[-0.18;0.43]	3 (1800)<br>SMD 0.15<br>[-0.19;0.48]	-	3 (579)<br>SMD 0.08<br>[-0.44;0.61]	.92

	I^2: 37%	I^2: 12%	I^2: 54%

Blank cells indicate no data available for variables.

*Significant at level 0.01

Results are represented as number of studies, number of participants, standardized mean differences (SMD) or risk ratios (RR), 95% Confidence interval and Restricted Maximum Likelihood method (I^2) statistics.

Table 7 The effect of integrated care on other outcomes.

Study	Intervention duration (months)	Knowledge	Process	Costs	Life style	Caregiver outcomes
[28]	24	-	-	The intervention costs were €324 per patient.<br><br>Excluding these costs, the intervention group had €584 (95% CI €86 to €1046) higher healthcare costs than did the usual care group and €645 (95% CI €28 to €1190) higher costs from the societal perspective.<br><br>Health outcomes were similar in both groups, except for 0.04 (95% CI −0.07 to −0.01) less QALYs in the intervention group.	-	-
[29]	12	-	-	-	The number of risk factors decreased significantly	-

					from baseline to six months in the immediate intervention group (MD=3.07, *p* = 0.003), but not in the waiting control<br><br>group (MD = -0.29, *p* = 0.77).	
[33]	18	Older adults with diabetes in the management group had significantly higher improvement of healthcare knowledge after intervention (MD 13.72; SD 9.85) than those in the control group (MD 1.30; SD 8.27) (*p* = .000).	-	-	Nonsignificant (*p* = .012) differences between the intervention (M change= 5.40, SD = 3.46) and control group (M=3.58, SD = 3.62) were found for diet measure.	-
[36]	4	-	Patients who received collaborative care had significantly higher scores for Patient Assessment Chronic Illness Care (PACIC) measure (intervention: 2.37 (SD 1.0); control 1.98 (SD 0.9)) and client satisfaction	-	-	-

			questionnaire (CSQ) (intervention MD = 2.90, SD=0.6; control MD=2.62, SD=0.6) as compared to those in the usual care group.			
[38]	2	-	-	-	-	Caregiver Strain Index score at eight weeks was significantly better in the intervention group (*p* < 0.0001), in comparison to the control group but with no difference at four weeks (*p* = 0.008).
[48]	3	-	-	-	-	In the experimental group at both post-test and 3 months after post-test, the caregiver burden measured with ZBI score (*p* = 0.001) was significantly lower than the control group, indicating a reduction in caregiver burden.
[55]	3	The diabetes self-	-	-	-	-

		management knowledge score improved by 2.56±2.72 (MD,SD) points in the experimental group and by 0.61±3.19 points in the control group, indicating statistically significant differences in scores between the experimental and control groups (*p* = .007).				
[56]	24	-	At the 12-month follow-up, there was no significant distinction observed (*p* = .31) between the intervention and control groups in terms of the patient. Assessment Chronic Illness Care (PACIC) measure.	-	-	-

Blank cells indicate no data available for variables.

Abbreviations: M, mean of the outcome assessment; CI, confidence interval; *p*, p-value; SD, standard deviation; MD, mean difference of the outcome assessment; QALY, quality-adjusted life year.

Note. The results in Table 7 above revealed that integrated care interventions had a positive impact on patient knowledge (2 studies; 168 participants) and caregiver outcomes (2 studies; 200 participants) as compared to control group. The effects of integrated care on life-style behaviour were reported in 2 studies (167 participants), and one of these studies reported a significant effect. Specifically, one of two studies showed a significant positive impact of integrated care on process-related outcomes as compared to the control group (see Table 6 in Appendix I). A cost-analysis of integrated care intervention for individuals with CRD was presented in 1 study (1,086 participants), showing that the intervention was not cost-effective as compared to the control group.

Table 8 Sensitivity analysis.

Primary outcomes

Outcome measures	All-cause mortality	All-cause hospital admissions	Adverse events	Healthcare use
**Risk of bias**				
Low	-	-	-	-
Unclear	-	-	-	SMD 0.66<br>[0.25;1.08]<br>I^2: -[a]
High	RR 0.60<br>[0.44;0.81]<br>I^2: 0%	RR 0.63<br>[0.56;0.71]<br>I^2: 7%	RR 0.53<br>[0.27;1.05]<br>I^2: 40%	SMD 0.11<br>[-0.76;0.98]<br>I^2: 0%
p	-	-	-	.01*
**Follow-up**				
Follow-up <12	RR 0.60<br>[0.44;0.81]<br>I^2: 0%	RR 0.63<br>[0.56;0.71]<br>I^2: 7%	RR 0.53<br>[0.27;1.05]<br>I^2: 40%	SMD 0.44<br>[-2.55;3.43]<br>I^2: 55%
Follow-up >=12	-	-	-	SMD 0.05<br>[-0.34;0.45]

				I^2: -[a]
p	-	-	-	.33
**Study size**				
Study size <200	RR 0.72<br>[0.10;5.24]<br>I^2: 0%	RR 0.57<br>[0.44;0.73]<br>I^2: 0%	RR 0.39<br>[0.27;0.57]<br>I^2: -[a]	SMD 0.30<br>[-0.50;1.10]<br>I^2: 57%
Study size >200	RR 0.53<br>[0.33;0.85]<br>I^2: 0%	RR 0.67<br>[0.57;0.78]<br>I^2: 0%	RR 0.62<br>[0.21;1.84]<br>I^2: 29%	-
p	.16	<.01*	.25	-

Blank cells indicate no data available for variables.

[a] Low number of studies

*Significant at level 0.01

Results are represented as number of studies, number of participants, standardized mean differences (SMD) or risk ratios (RR), 95% Confidence interval and Restricted Maximum Likelihood method (I^2) statistics.

Note.

There was limited indication that variations in intervention duration or study settings had different effects on healthcare use ($p = .33$), quality of life ($p = .48$), physical functioning ($p = .21$), weight management ($p = .37$), self-management ($p = .34$), mental health ($p = .26$), blood pressure control ($p = .89$), pulmonary measures ($p = .74$) (study settings p-values: all-cause mortality $p = .83$; all-cause hospital admissions $p = .70$; healthcare use $p = .10$; quality of life $p = .18$; physical functioning $p = .08$; weight management $p = .93$; mental health $p = .11$; self-management $p = .45$; cholesterol $p = .60$, pulmonary measures $p = .73$, blood pressure control $p = .35$). However, differences in effect were found for adverse events ($p < .01$) and HbA1c ($p < .01$) based on study setting. Considering that there was only one grouping based on duration, no differences were observed in outcomes all-cause mortality, all-cause hospital admissions, adverse events, cholesterol, and HbA1c.

The results of sensitivity analysis showed that restricting analyses to studies with lower risks of bias provided no different treatment effects for quality of life ($p = .71$), physical functioning ($p = .92$), weight management ($p = .20$), mental health ($p = .99$), self-management ($p = .33$), HbA1c ($p = .28$), cholesterol ($p = .20$), pulmonary measures ($p = .73$). The risk of bias resulted in different results for healthcare use ($p = .01$) and blood pressure control ($p < .01$; see Table 8 above). We found no effect of sample size for all-cause mortality ($p = .16$), adverse events ($p = .25$), quality of life ($p = .14$), physical functioning (p=.17), weight management ($p = .92$), mental health ($p = .17$), HbA1c ($p = .74$) cholesterol ($p = .44$), and pulmonary measures ($p = .73$; Table 7 in Appendixes). However, sample size resulted in significant different results for all-cause hospital admissions ($p < .01$), self-management ($p < .01$), and blood pressure control ($p < .01$).

Additionally, our analysis also showed that restricting analyses to studies with follow-up <12 months provided no different treatment effects for healthcare use ($p = .33$), quality of life ($p = .92$), physical functioning ($p = .47$), weight management ($p = .92$), mental health ($p = .50$), HbA1c ($p = .47$), cholesterol ($p = .55$), and blood pressure control ($p = .15$; Table 7 in Appendix I). The follow-up of less than 12 months resulted only in different results for pulmonary measures ($p = .02$).

Table 9 Secondary outcomes

**Outcome measures**	**Quality of life**	**Physical functioning**	**Weight management**	**Mental health**	**Self- management**
**Risk of bias**					
Low	SMD 0.53	SMD 0.16	SMD 0.19	SMD 0.30	SMD 0.59
	[-4.88;5.95]	[-0.23;0.54]	[-0.22;0.60]	[-0.25;0.85]	[-0.41;1.58]
	I^2: 86%	I^2: 72%	I^2: 65%	I^2: 86%	I^2: 62%
Unclear	-	-	-	SMD 0.24	SMD 0.11
				[-0.05;0.54]	[-0.29;0.52]
				I^2: -[a]	I^2: -[a]
High	SMD 0.36	SMD 0.17	SMD 0.01	SMD 0.29	SMD 0.24
	[-0.03;0.76]	[0.02;0.33]	[-0.17;0.18]	[0.11;0.47]	[0.01;0.47]

	I^2: 89%	I^2: 68%	I^2: 0%	I^2: 80%	I^2: 74%
p	.71	.92	.20	.99	.33
**Follow-up**					
Follow-up <12	SMD 0.38<br>[0.00;0.76]<br>I^2: 88%	SMD 0.16<br>[0.01;0.32]<br>I^2: 71%	SMD 0.05<br>[-0.13;0.24]<br>I^2: 60%	SMD 0.30<br>[0.11;0.48]<br>I^2: 82%	SMD 0.30<br>[0.10;0.50]<br>I^2: 74%
Follow-up >=12	SMD 0.45<br>[0.05;0.85]<br>I^2: -[a]	SMD 0.23<br>[-0.52;0.98]<br>I^2: 0%	SMD 0.20<br>[-1.04;1.45]<br>I^2: 0%	SMD 0.23<br>[-0.20;0.67]<br>I^2: 0%	-
p	.92	.47	.24	.50	-
**Study size**					

Study size <200	SMD 0.60<br>[-0.09;1.29]<br>I^2: 88%	SMD 0.22<br>[0.04;0.41]<br>I^2: 63%	SMD 0.07<br>[-0.04;0.18]<br>I^2: 0%	SMD 0.34<br>[0.13;0.55]<br>I^2: 73%	SMD 0.45<br>[0.23;0.67]<br>I^2: 45%
Study size >200	SMD 0.16<br>[-0.01;0.33]<br>I^2: 87%	SMD 0.06<br>[-0.16;0.28]<br>I^2: 74%	SMD 0.09<br>[-0.79;0.98]<br>I^2: 88%	SMD 0.19<br>[-0.17;0.54]<br>I^2: 88%	SMD 0.04<br>[-0.26;0.34]<br>I^2: 61%
p	.14	.17	.92	.17	<.01*

Blank cells indicate no data available for variables.

[a] Low number of studies

*Significant at level 0.01

Results are represented as number of studies, number of participants, standardized mean differences (SMD) or risk ratios (RR), 95% Confidence interval and Restricted Maximum Likelihood method (I^2) statistics.

Table 10 Tertiary outcomes

Outcome measures	HbA1c	Cholesterol	Pulmonary measures	Blood pressure control
**Risk of bias**				
Low	SMD 0.15<br>[-0.57;0.87]<br>I^2: 48%	SMD 0.03<br>[-0.42;0.48]<br>I^2: 0%	SMD 0.11<br>[-0.20;0.43]<br>I^2: -[a]	SMD 0.33<br>[-0.25;0.90]<br>I^2: 0%
Unclear	SMD 0.56<br>[0.26;0.87]<br>I^2: -[a]	-	-	-
High	SMD 0.53<br>[0.13;0.93]	SMD -0.05<br>[-0.22;0.12]	SMD 0.21<br>[-0.42;0.85]	SMD 0.08<br>[-0.02;0.17]

	I^2: 89%	I^2: 0%	I^2: 66%	I^2: 12%
p	.28	.20	.73	<.01*
**Follow-up**				
Follow-up <12	SMD 0.48<br>[0.16;0.80]<br>I^2: 87%	SMD -0.04<br>[-0.15;0.07]<br>I^2: 0%	SMD 0.07<br>[-0.06;0.20]<br>I^2: 0%	SMD 0.09<br>[-0.02;0.19]<br>I^2: 25%
Follow-up >=12	SMD 0.08<br>[-0.27;0.43]<br>I^2: -[a]	SMD 0.07<br>[-0.28;0.42]<br>I^2: -[a]	SMD 0.57<br>[0.15;0.99]<br>I^2: -[a]	SMD 0.27<br>[-1.23;1.77]<br>I^2: 0%
p	.47	.55	.02**	.15
**Study size**				

Study size <200	SMD 0.44<br>[0.12;0.77]<br>I^2: 87%	SMD 0.02<br>[-0.10;0.13]<br>I^2: 0%	SMD 0.11<br>[-0.20;0.43]<br>I^2: -[a]	SMD 0.26<br>[0.14;0.38]<br>I^2: 0%
Study size >200	SMD 0.63<br>[0.30;0.95]<br>I^2: -[a]	SMD -0.06<br>[-1.18;1.07]<br>I^2: 0%	SMD 0.21<br>[-0.42;0.85]<br>I^2: 66%	SMD 0.05<br>[-0.12;0.21]<br>I^2: 29%
p	.74	.44	.73	<.01*

Blank cells indicate no data available for variables.

[a] Low number of studies

*Significant at level 0.01

**Significant at level 0.05

Results are represented as number of studies, number of participants, standardized mean differences (SMD) or risk ratios (RR), 95% Confidence interval and Restricted Maximum Likelihood method (I^2) statistics.

Appendix II Tertiary outcomes

HbA1c

Integrated care interventions had a moderate positive effect on HbA1c levels, as reported in 15 studies involving 1,812 participants (SMD, 0.45; 95% CI, 0.15 to 0.75) for CVD (1 study; 124 participants; SMD 0.08; 95% CI, -0.27 to 0.43), DMT2 (9 studies; 943 participants; SMD 0.36; 95% CI, 0.10 to 0.62), and multiple comorbidities (5 studies; 745 participants; SMD 0.69; 95% CI, -0.32 to 1.69). However, the reviewed studies were characterised by a high level of heterogeneity (I2 = 87%; Figure 1 below). No substantial evidence indicated that different types of integrated care interventions had differing effects on HbA1c levels (patient empowerment, SMD 0.43; 95% CI, 0.10 to 0.75; network care coordination, SMD 0.48; 95% CI, -0.10 to 1.06; $p_{\text{subgroup difference}} = 0.85$). The quality of the evidence for HbA1c was graded as low (Table 2 in the main text). The results of our analysis for each disease group showed that integrated care had a positive effect on HbA1c levels for people with DMT2 and multiple comorbidities, while no such evidence was found for CVD (Table 6 in Appendix I).

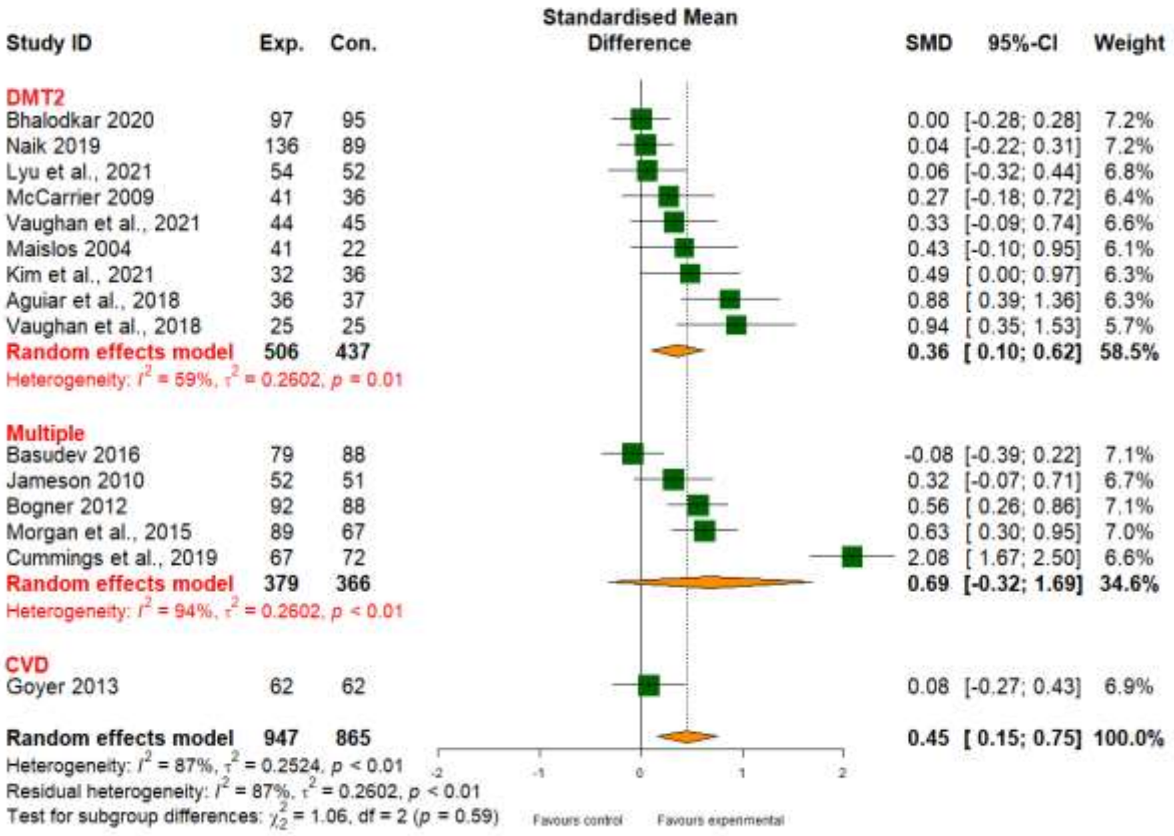

Figure 1 Effect of integrated care on HbA1c.

Cholesterol

In 12 studies that involved 966 participants, the impact of integrated care on cholesterol levels was uncertain (6 studies; 966 participants; SMD -0.02; 95% CI, -0.12 to 0.07) for CVD (1 study; 124 participants; SMD 0.07; 95% CI, -0.28 to 0.42), DMT2 (3 studies; 428 participants; SMD 0.01; 95% CI, -0.17 to 0.20), and multiple comorbidities (2 studies; 414 participants; SMD -0.08; 95% CI, -1.00 to 0.83), as there was no variation in the results among the reviewed studies (I2 = 0%; Figure 2 below). We found no indication that different types of integrated care interventions had differing effects on cholesterol levels (patient empowerment, SMD 0.03; 95% CI, -0.21 to 0.27; network care coordination, SMD -0.04; 95% CI, -0.29 to 0.20; $p_{\text{subgroup difference}}$ = 0.39). The quality of the evidence for cholesterol was low (Table 2 in the main text).

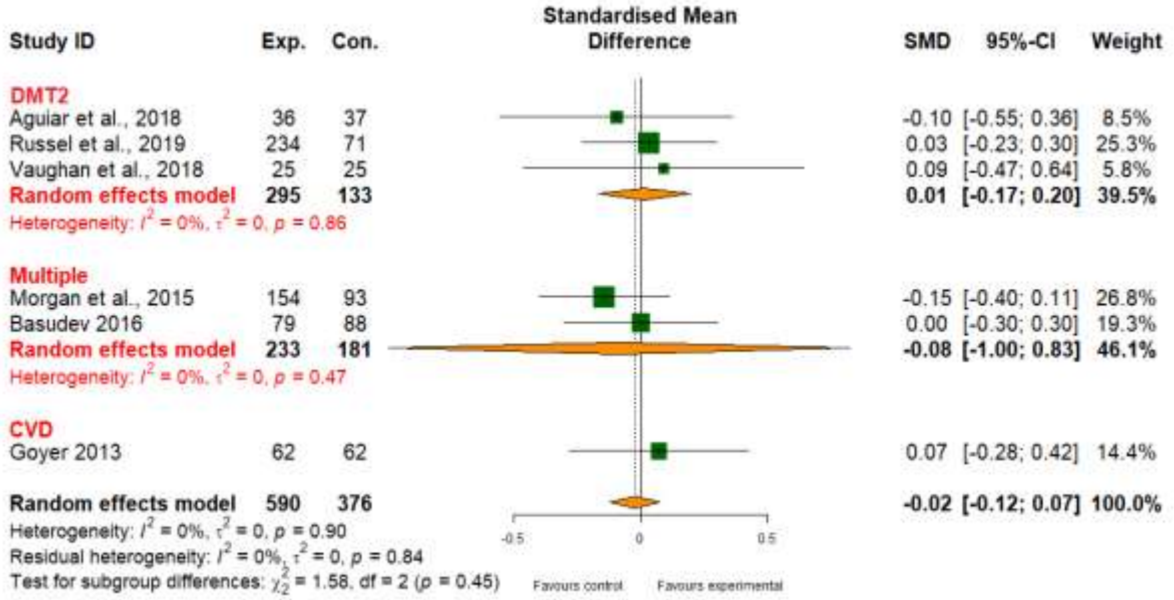

Figure 2 Effect of integrated care on cholesterol.

Pulmonary measures

According to 4 studies involving 1,498 participants, integrated care interventions did not have a significant impact on pulmonary functioning as compared to standard care management (SMD, 0.17; 95% CI, -0.16 to 0.49); variation among the reviewed studies was moderate (I2 = 48%) for CRD (Figure 3 below). The quality of evidence for pulmonary functioning was rated as low (Table 2 in the main text).

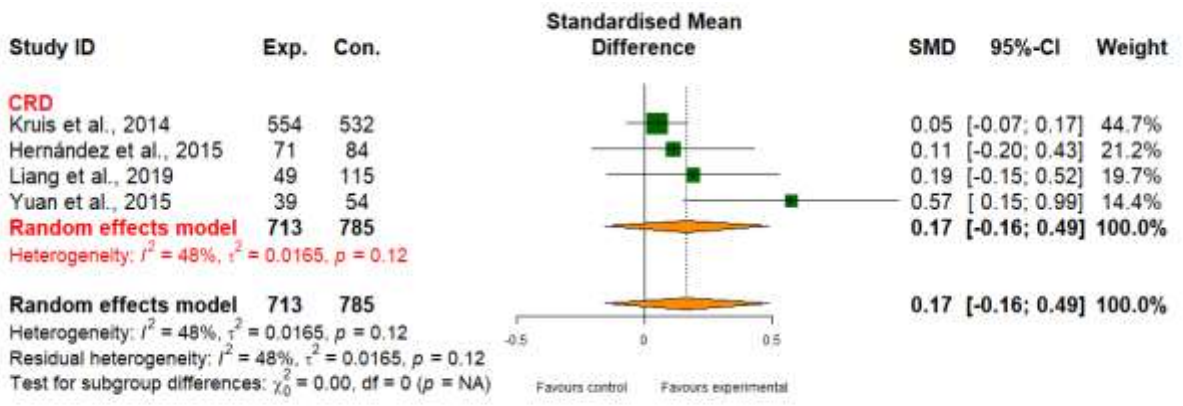

Figure 3 Effect of integrated care on pulmonary measures.

Blood pressure control

Integrated care was not found to significantly improve the blood pressure control (11 studies; 2,996 participants; SMD, 0.11; 95% CI, 0.00 to 0.21) for CVD (3 studies; 1,800 participants; SMD, 0.15; 95% CI, -0.19 to 0.48), DMT2 (5 studies; 617 participants; SMD, 0.13; 95% CI, -0.18 to 0.43), and multiple comorbidities (3 studies; 579 participants; SMD, 0.08; 95% CI, -0.44 to 0.61). The level of heterogeneity among the reviewed studies was low (I2=24%; Figure 4 below). The effects of different types of interventions did not reach statistical significance (patient empowerment, SMD 0.17; 95% CI, 0.01 to 0.32; network care coordination, SMD 0.04; 95% CI, -0.19 to 0.27; $p_{\text{subgroup difference}}$ = 0.21). The quality of the evidence for blood pressure control was moderate (Table 2 in the main text)..

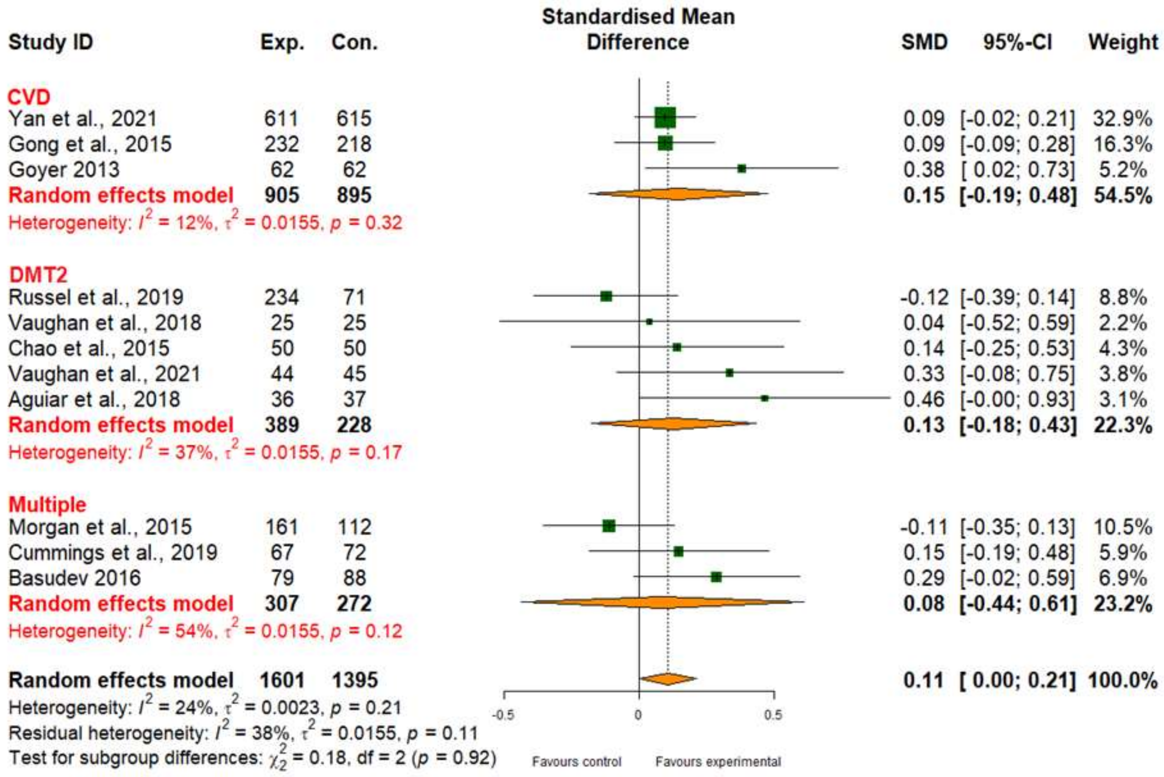

Figure 4 Effect of integrated care on blood pressure control

Appendix III

Note: There was little evidence of funnel plot asymmetry in treatment effects for hospital admissions, adverse events, healthcare use, quality of life, mental health, weigh management, cholesterol, HbA1c, pulmonary measures, and blood pressure control (Figures 5-17 in Appendix I). However, we found evidence of funnel plot asymmetry in treatment effects for mortality, physical functioning, and self-management ($p < .05$) (Figures 5-17 in Appendix III).

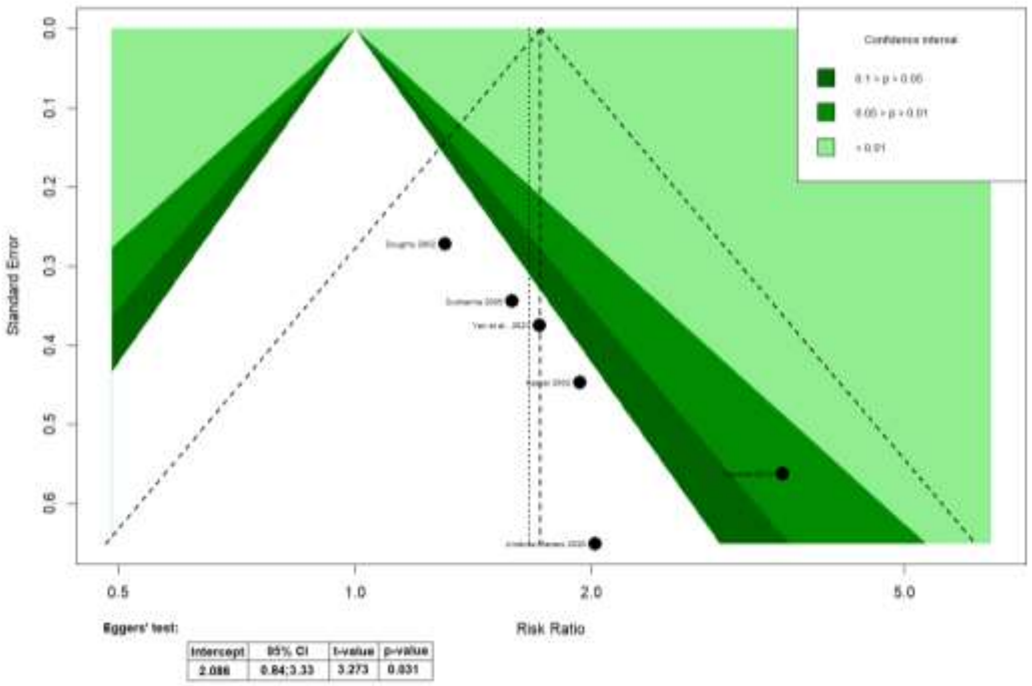

Figure 5 Funnel plot all-cause mortality.

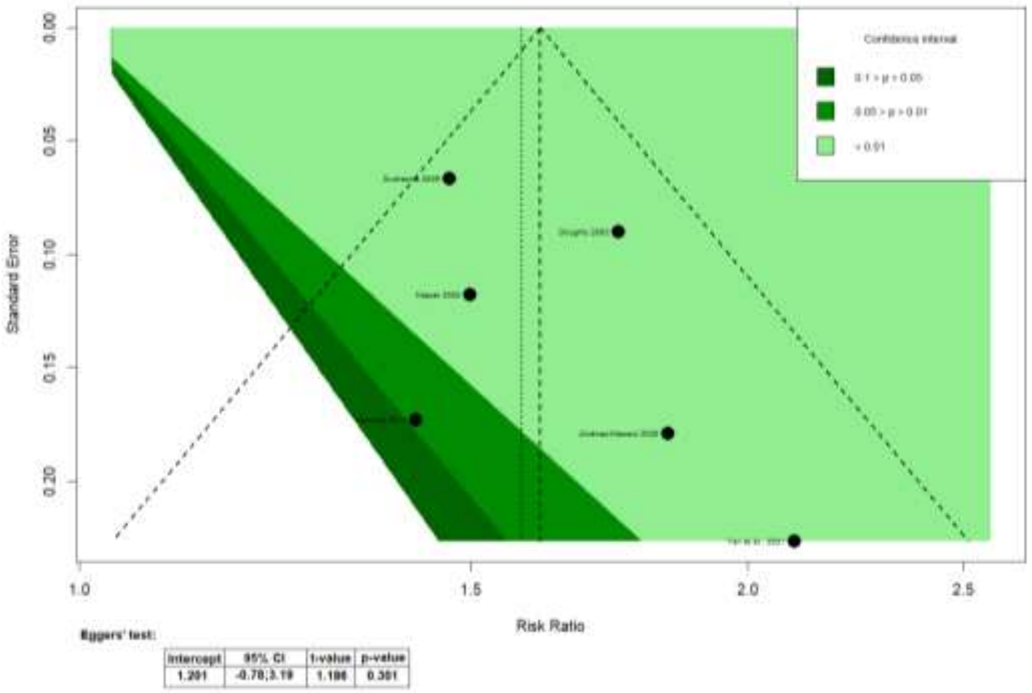

Figure 6 Funnel plot all-cause hospitalization.

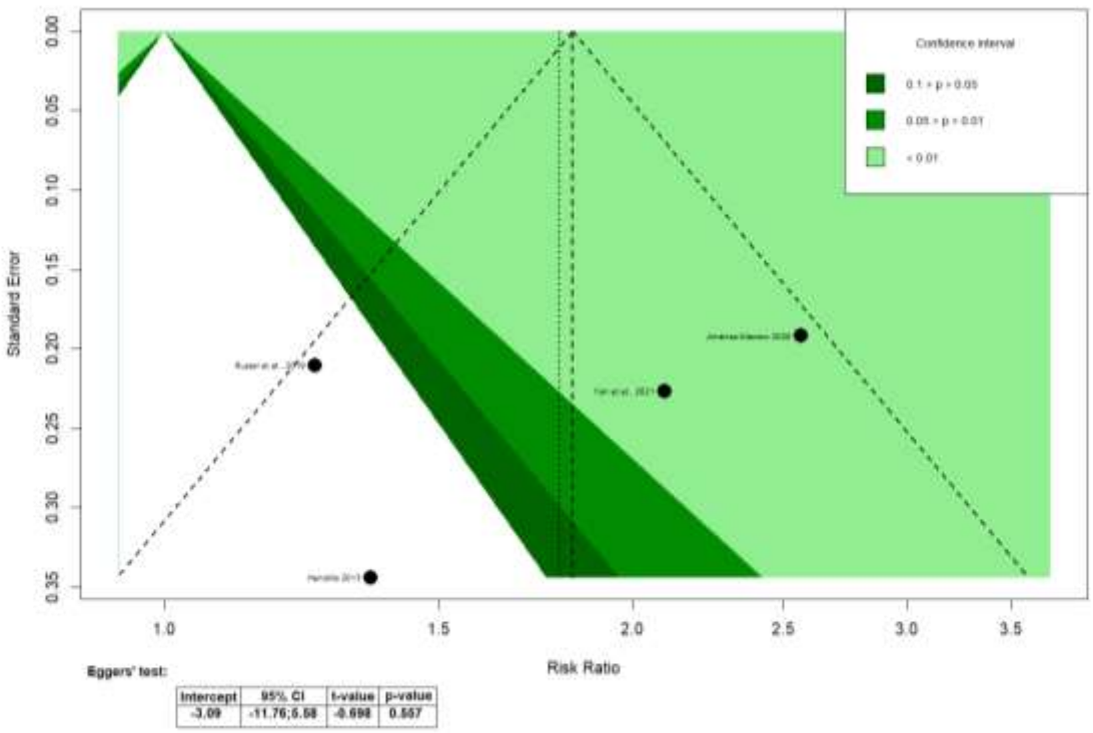

Figure 7 Funnel plot adverse events.

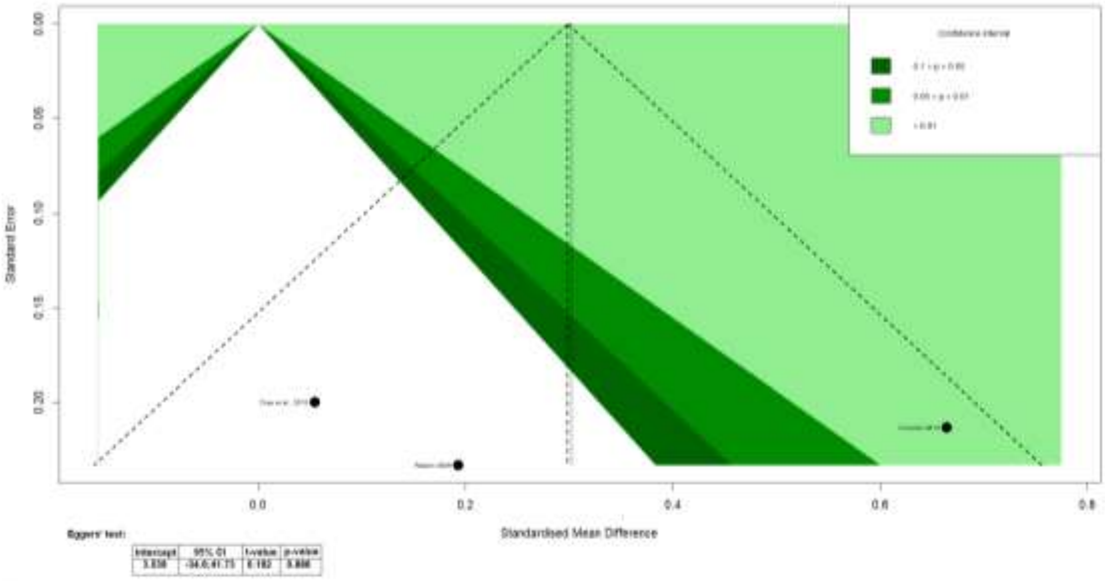

Figure 8 Funnel plot healthcare use.

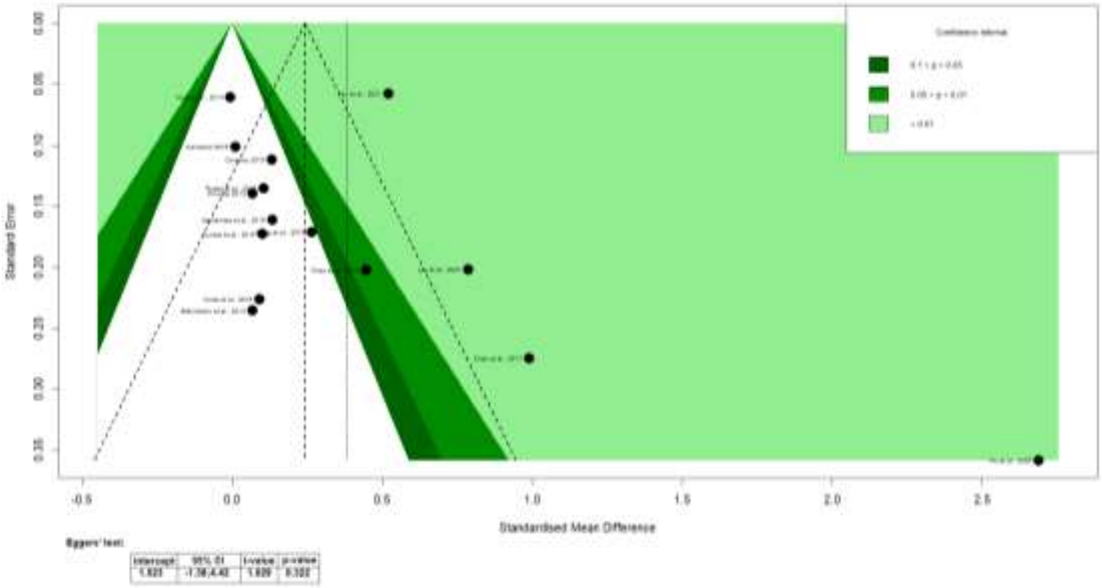

Figure 9 Funnel plot quality of life.

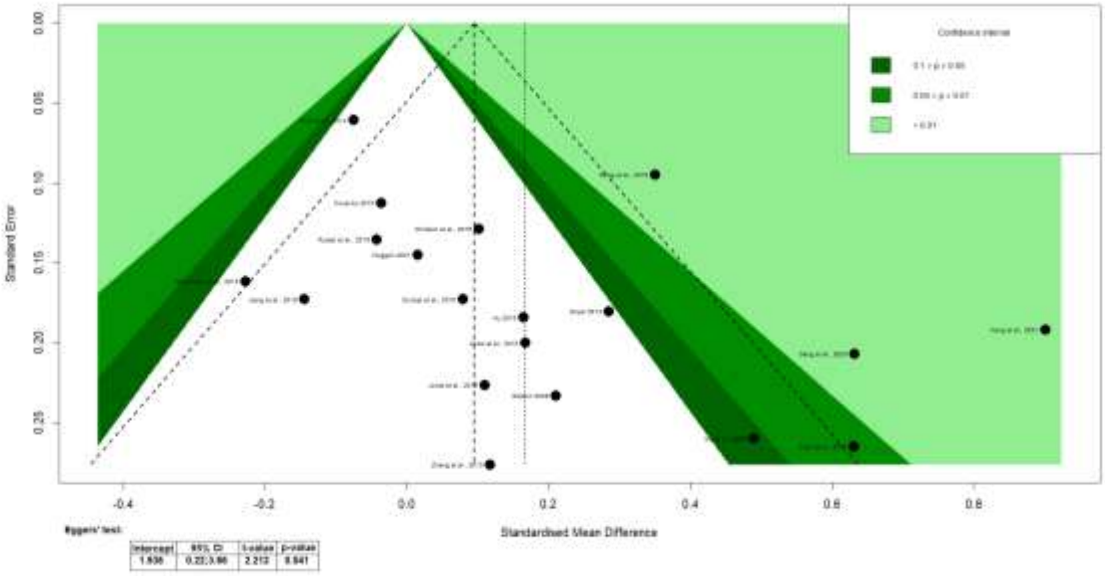

Figure 10 Funnel plot physical functioning.

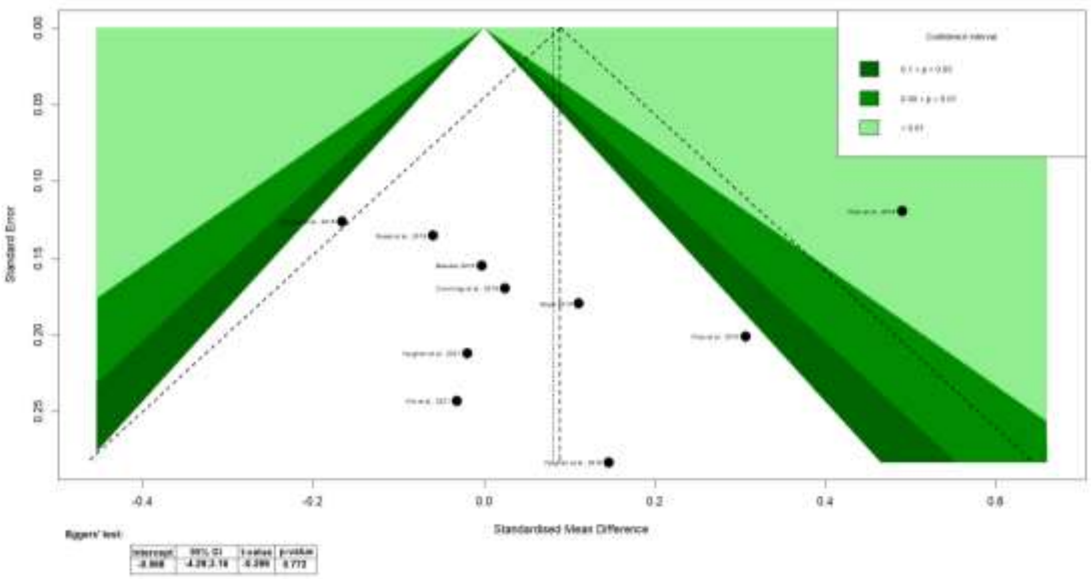

Figure 11 Funnel plot weight management.

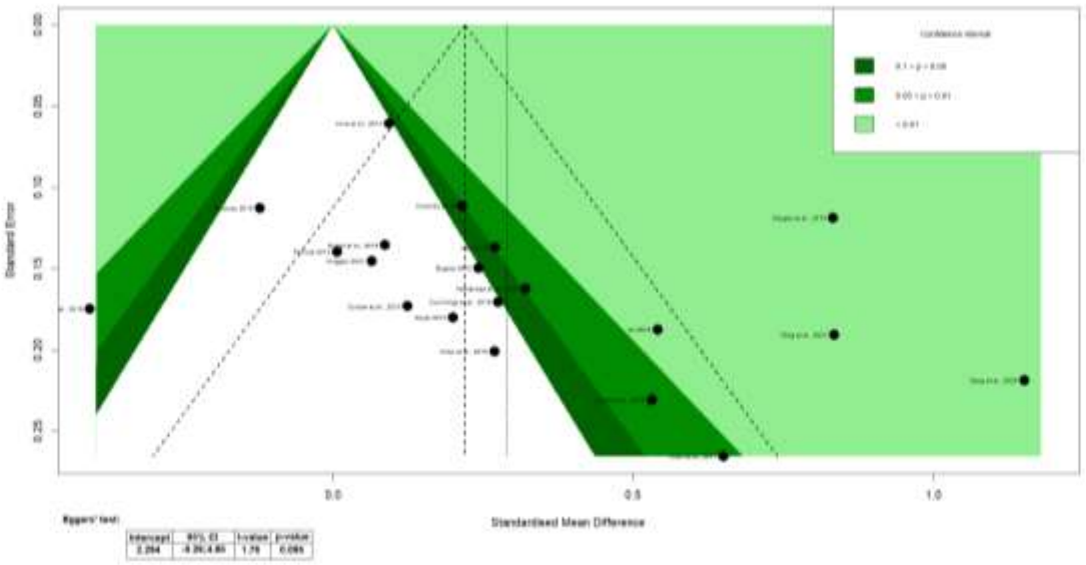

Figure 12 Funnel plot mental health.
